# Extension of the π-system of monoaryl-substituted norbornadienes with acetylene bridges: influence on the photochemical conversion and storage of light energy

**DOI:** 10.3762/bjoc.20.254

**Published:** 2024-11-21

**Authors:** Robin Schulte, Dustin Schade, Thomas Paululat, Till J B Zähringer, Christoph Kerzig, Heiko Ihmels

**Affiliations:** 1 Department of Chemistry-Biology, Center of Micro- and Nanochemistry and (Bio-)Technology (Cµ), University of Siegen, Adolf-Reichwein-Str. 2, 57068 Siegen, Germany; 2 Department of Chemistry, Johannes Gutenberg University Mainz, Duesbergweg 10–14, 55128 Mainz, Germany

**Keywords:** light energy conversion, photochemistry, photochromism, quadricyclanes, Sonogashira–Hagihara coupling

## Abstract

The photochromic norbornadiene/quadricyclane pair is a promising system for molecular solar thermal (MOST) energy storage, with which solar energy may be converted, stored, and released as heat in one integral molecular system. Herein, we present the synthesis of mono-, bis-, and tris-norbornadiene derivatives with alkynylbenzene and alkynylnaphthalene core units, along with studies of their photochemical properties. The target compounds were synthesized by Sonogashira–Hagihara coupling reactions of 2-bromonorbornadiene and the corresponding arylacetylenes. The norbornadienes showed absorption maxima in the range of 310–345 nm and long-wavelength zero onsets of up to 420 nm. The photoisomerization quantum yields were as high as 59% per photoisomerization event and the resulting quadricyclanes showed half-lives of up to 8 h at room temperature. Furthermore, the norbornadienes were transformed quantitatively into their quadricyclane photoproducts by irradiation with green light (520 nm) in the presence of a photosensitizer.

## Introduction

**Scheme 1:**
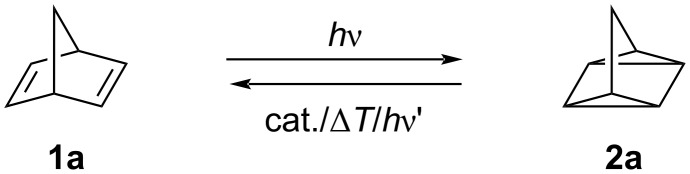
Photoinduced [2 + 2]-cycloaddition–cycloreversion cycle of norbornadiene (**1a**) and quadricyclane (**2a**).

**Figure 1:**
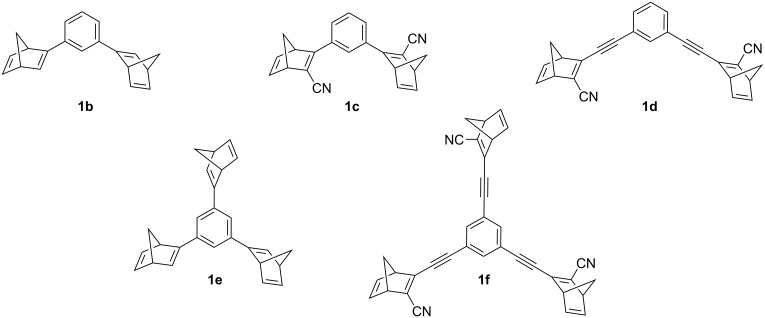
Representative bis- and tris-norbornadienyl-substituted benzene derivatives.

The application of sustainable energy storage and supply has become a very important issue both from an economic and ecological point of view. In particular, the global energy demand is continuously expanding because of the increasing population, the development of new, energy-intensive technologies and overconsumption of energy [1–2]. In this context, windpower plants and solar photovoltaic systems have become increasingly important contributions to the overall available energy, although both technologies still pose some problems regarding the storage of the converted energy [3–8]. This drawback may be solved with the progress in battery technology, however, the use of high-performance batteries still requires some problematic materials, which are difficult to access, hardly recyclable, or which may harm the environment [9]. Thus, in the present situation, the efficient and flexible conversion and storage of energy requires the variable combination of different, complementary storage systems, and as a consequence, the development of alternative methods for energy storage is still an important task to contribute to the mix of storage systems. One promising approach towards storage of solar energy is represented by the molecular solar thermal (MOST) energy storage systems, which have the ability to convert and store (sun)light energy in reversible photochemical reactions. Specifically, this approach is based on photoactive compounds that are converted into photoisomers with higher energy upon absorption of light. In turn, this reaction is reversible, thus allowing to release the stored chemical energy as heat in a specifically triggered back reaction [7–8,10–13]. For a practicable MOST system, some key parameters have to be ensured, such as absorption in the range of the solar spectrum, a high quantum yield of the photoreaction, long half-lives of the photoproducts, and a high energy storage density [7–8,12–14]. In this context, several different MOST systems have been explored, including, for example, anthracenes [15], azobenzenes [16–19], dihydroazulenes [20–21], or azaborines [22–23]. However, the most prominent feasible MOST system is the [2 + 2] photocycloaddition–cycloreversion cycle between norbornadiene (**1a**) and quadricyclane (**2a**), which has several advantageous properties (Scheme 1) [8,14,24]. In particular, this system shows a high energy storage capacity of up to 1 MJ/kg, and the energy can be conveniently released in form of heat by controlled photolysis, thermolysis, or catalysis [8,14]. However, the parent norbornadiene (**1a**) only absorbs ultraviolet light, which is of limited availability in sunlight. Furthermore, the photoisomerization quantum yield is very low and therefore not suitable for an application [7–8,14]. One promising way to overcome these disadvantages is the introduction of a donor–acceptor system at the norbornadiene double bonds, which has resulted in the development of new generations of modified norbornadiene derivatives with diverse variations of donor and acceptor substituents [25–32]. However, such a modification often leads to higher molar masses and thus to lower energy densities [7,25,33]. Consequently, further fine tuning of the substituents is necessary to accomplish a reasonable balance between the MOST parameters. Hence, to increase the energy density and to induce a decent red shift of the absorption, two, three or even more norbornadiene units may be combined in one integral molecule by attachment to a shared, linking arene unit, such as realized in bis- and tris-norbornadienes **1b**–**f** (Figure 1) [7,12,30,34–35]. In this particular case, derivatives with only one substituent at the norbornadiene double bond exhibited favorable storage properties because of a lower molecular weight, however, their absorption still lies in the UV range [30]. Therefore, we aimed at further modification of the core structure of bis-and tris-norbornadienes, which may lead to a more pronounced red shift of the absorption while maintaining the high energy density. As this has been shown in other cases already [34], we anticipated that the integration of an acetylene bridge between the arene core and the norbornadiene units in compounds **1h**–**n** may induce these effects. Herein, we present the synthesis of these target molecules and our studies of their photochemical and physicochemical properties with a focus on energy conversion and storage.

## Results and Discussion

**Scheme 2:**
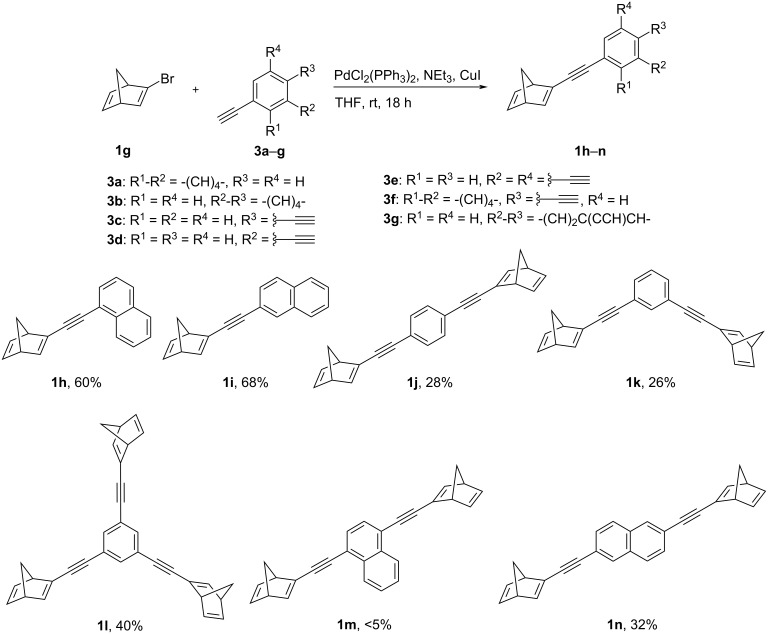
Synthesis of alkynyl-arene-linked norbornadienes **1h**–**n** by Sonogashira–Hagihara coupling reactions.

The norbornadiene derivatives **1h**–**l**,**n** were synthesized by Sonogashira–Hagihara coupling reaction of 2-bromonorbornadiene (**1g**) [36] with the corresponding arylacetylenes **3a**–**g** (Scheme 2). As an exception, 1,4-bis-norbornadienylnaphthalene (**1m**) could not be obtained by this route because the product could not be separated from the complex product mixture. The monosubstituted derivatives **1h** and **1i** were isolated in good yields (60–68%), whereas the bis- and tris-substituted derivatives **1j**–**l**,**n** were only obtained in low yields (26–40%). The latter were more difficult to isolate because by-products interfered with the chromatographic separation; however, these yields are similar to the ones reported for resembling norbornadienes [34,37]. The novel compounds **1h**–**l**,**n** were identified and fully characterized by NMR spectroscopy (^1^H, ^13^C, COSY, HSQC, HMBC), melting point, and elemental analysis. All products showed the characteristic ^1^H NMR spectroscopic signals of norbornadienes, in particular two signals at ca. 2.10 ppm and 2.20 ppm (7-CH_2_) and two broad singlets between 3 and 4 ppm (bridgehead 1- and 4-CH). In addition, typical ^13^C NMR shifts of the aryl-substituted acetylenes at ca. 85 ppm and 95 ppm were observed. The norbornadienes **1h**–**l**,**n** exhibit very good solubility in organic solvents, like ethyl acetate, chloroform, or benzene, whereas they have poor solubility in cyclohexane, acetonitrile, or ethanol. In polar protic solvents, like methanol or water, they are insoluble.

The absorption spectra of compounds **1h**–**l**,**n** were recorded in different solvents (Table 1, cf., Supporting Information File 1, Figure S1). In general, the employed solvents had only a marginal influence on the absorption properties of each compound. With slight variations caused by the different substitution patterns, the compounds **1h**–**l**,**n** showed the characteristic long-wavelength bands with maxima between 310 nm (**1k**) and 345 nm (**1n**) and low-energy zero onsets from 345 nm (**1k**) to 420 nm (**1n**). Hence, the red shift of the absorption bands correlates with the size of the respective π-system of the derivatives, and is, thus, more pronounced for the naphthalene-bridged compounds. Likewise, the increased size of the conjugated π-system also led to a red shift of the absorption bands in comparison with the ones of the corresponding norbornadiene derivatives without acetylene bridges (**1b**,**e**) [29–30]. Accordingly, a slightly more pronounced red shift (20 nm) has been reported for derivatives **1d** and **1f** which carry an additional electron-acceptor in the 3-position of the norbornadiene unit [33–34].

**Scheme 3:**
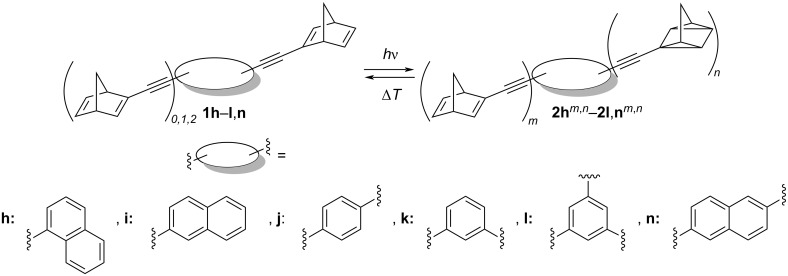
Photoisomerization of norbornadiene derivatives **1h**–**l**,**n** (20 µM) to quadricyclanes **2h**–**l**,**n** in cyclohexane**,** *m* = number of norbornadienylethynyl units, *n* = number of quadricyclylethynyl units.

**Figure 2:**
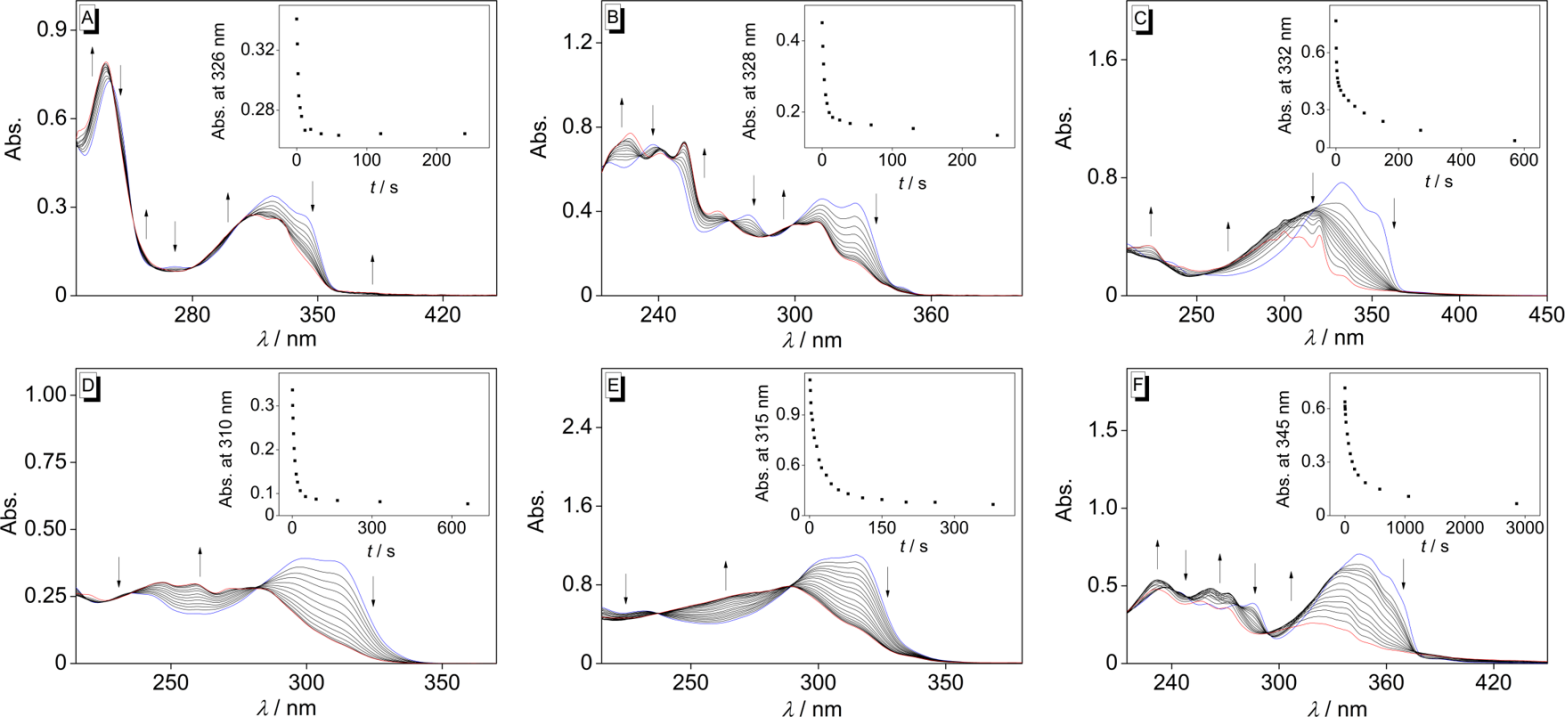
Photometric monitoring of the irradiation of **1h** (A), **1i** (B), **1j** (C), **1k** (D), **1l** (E), and **1n** (F); λ_ex_ = 315 nm (**1h**, **1i**, **1k**, and **1l**) or 340 nm (**1j**, **1n**). Insets: plot of absorption at long-wavelength maximum versus irradiation time *t*.

The photoisomerization of the norbornadiene derivatives **1h**–**l**,**n** (Scheme 3) was investigated photometrically in cyclohexane (20 µM, Figure 2), because the norbornadienes and the corresponding quadricyclanes are sufficiently soluble in this solvent and because it allows direct comparison with literature data [30,34]. As a general trend, the long-wavelength absorption maximum of each substrate decreased for all derivatives during the photoreaction, whereas the blue-shifted maximum of the photoproduct was formed. In addition, isosbestic points were observed for the derivatives **1h** (A), **1i** (B), **1k** (D), and **1l** (E). The irradiations of the derivatives **1h**, **1i**, **1k**, and **1l** were stopped once no more changes of the absorbance spectra were observed. In the case of compounds **1j** and **1n**, the endpoint of the photoreaction could not be identified, as a steady decrease of the whole absorption spectra during irradiation indicated secondary decomposition reactions.

**Scheme 4:**
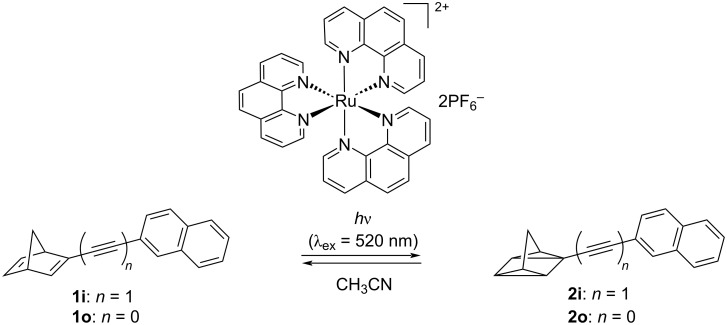
Triplet-sensitized photoisomerization of norbornadiene **1o** to quadricyclane **2o**.

We previously reported triplet energy transfer between ^3^[Ru(phen)_3_](PF_6_)_2_ and the corresponding arylnorbornadienes without an acetylene bridge, such as **1o**, leading to efficient and quantitative conversion to the quadricylanes (Scheme 4) [38]. Hence, we assumed that triplet-sensitized isomerization of the mono-, bis-, and tris-norbornadiene derivatives with an alkynylbenzene and alkynylnaphthalene core is also feasible. Quenching experiments of [Ru(phen)_3_](PF_6_)_2_ (triplet energy *E*_T_ = 2.19 eV [39]) with **1i** in acetonitrile yielded a triplet energy transfer rate of *k*_EnT_ = 5.8 × 10^9^ M^−1^ s^−1^ indicating very efficient deactivation of the triplet sensitizer (Figure S4 in Supporting Information File 1). This rate is approximately an order of magnitude faster compared with the corresponding naphthylnorbornadiene **1o** without the acetylene bridge, which has a reported triplet energy of 2.41 eV (for details, see Supporting Information File 1, chapter S7). These results suggest that the triplet energy of **1i** is significantly lower than 2.4 eV and even triplet sensitizers with lower triplet energies than that of [Ru(phen)_3_](PF_6_)_2_ could be employed for this reaction, which might even allow red light-driven switching.

**Figure 3:**
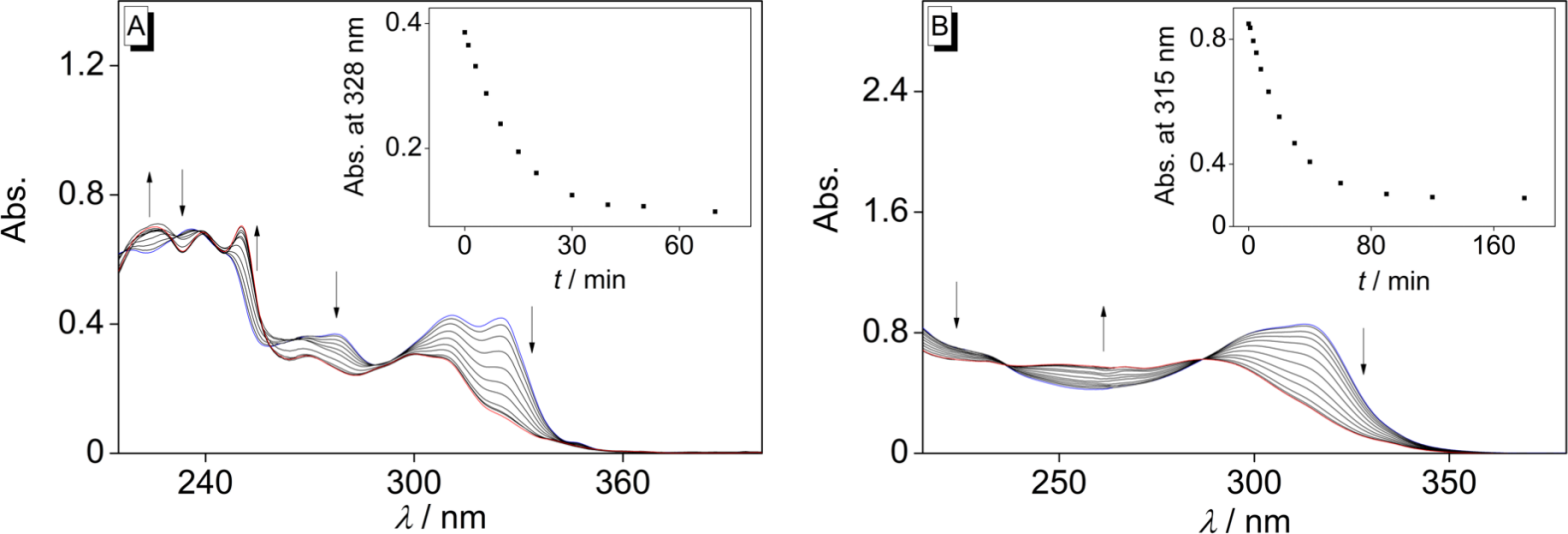
Photometric monitoring of the irradiation of **1i** (A) and **1l** (B) in the presence of [Ru(phen)_3_](PF_6_)_2_; λ_ex_ = 520 nm. For clarity, the absorption of the sensitizer has been deducted. Insets: plot of absorption at long-wavelength maximum versus irradiation time *t* [38].

In addition to these kinetic studies, the photoisomerization reactions of **1i** and **1l** (20 µM) were investigated in the presence of [Ru(phen)_3_](PF_6_)_2_ (20 µM) at λ_ex_ = 520 nm in acetonitrile (Scheme 4). This solvent was required because of the solubility of the sensitizer. Under these conditions, both derivatives **1i** and **1l** showed the same development of absorption spectra during irradiation as upon direct irradiation, although the reaction times were significantly higher, most likely because of the much weaker molar absorption coefficient of [Ru(phen)_3_](PF_6_)_2_ at 520 nm (≈900 M^−1^ cm^−1^), and the fact that the photosensitization process is bimolecular (Figure 3). Indeed, the quenching efficiency of triplet-excited [Ru(phen)_3_](PF_6_)_2_ by **1i** was calculated to be as low as 5% under the highly diluted conditions required for the experiments in Figure 3.

In order to gain structural information about the photoproducts, the photoisomerization reactions were also examined by ^1^H NMR spectroscopy. Initially, all derivatives showed the isomerization reactions to the corresponding quadricyclane derivatives, as identified by the characteristic ^1^H NMR signal pattern of the quadricyclane fragment with five multiplets at around 1.6 ppm, 1.7 ppm, 1.8 ppm, 2.1 ppm, and 2.2 ppm. However, after relatively short irradiation times of only 30 min, additional signals were detected in the spectrum, which could not be assigned to distinct by-products or secondary products. Unfortunately, the NMR signals of the by-products overlapped with the ones of the norbornadiene and the quadricyclane signals, so that the quantitative analysis of the photostationary state was not possible with NMR spectroscopy. These observations are surprising, since the 3-acceptor-substituted derivatives and the ones without acetylene linkers have been reported to be converted quantitatively into their corresponding quadricyclanes [24,29–30,34].

As the photometric analysis of the photosensitized reaction of **1i** and **1l** pointed to a relatively clean and robust reaction, the irradiation of **1i** in the presence of [Ru(phen)_3_](PF_6_)_2_ at λ_ex_ = 520 nm was additionally monitored by in situ NMR spectroscopy [40] (Figure S41 in Supporting Information File 1). With this experiment, it was shown unambiguously that **1i** was quantitatively converted into the corresponding quadricyclane **2i** in the presence of the triplet sensitizer, as clearly indicated by the exclusive formation of the NMR signals of the photoproduct **2a** at 1.60–1.63 ppm, 1.69–1.71 ppm, 1.82–1.85 ppm, 2.07–2.11 ppm, and 2.21–2.25 ppm. Thus, it is possible to convert this monosubstituted norbornadiene efficiently into the corresponding quadricyclane with visible light, and thus also by sunlight. Unfortunately, the derivatives **1h** and **1j**–**l**,**n** could not be investigated in the presence of the photosensitizer because these substrates have no sufficient solubility in acetonitrile.

To assess the efficiency of the photoreactions, photoisomerization quantum yields were determined in cyclohexane by actinometry (λ_ex_ = 310 nm or 340 nm) [41–42]. The quantum yields varied from only 1% per conversion event, i.e. per norbornadiene unit, for the naphthyl-substituted derivative **1n** to 59% for the *m*-phenyl-linked derivative **1k** (Table 1). In general, the derivatives **1h**–**l**,**n** showed significantly lower quantum yields than the 3-cyano-substituted derivatives **1d** and **1f** and the analogues **1c** and **1e** without an alkyne linker [29–30,34]. As the only exception, the *m*-substituted derivative **1k** has a higher quantum yield than its analog **1b**, which is, however, still lower than the one of the cyano-substituted norbornadiene **1c** [30,34]. In addition, substitution at the 2- and 2,6-position of the naphthalene unit resulted in low quantum yields, whereas the attachment of the norbornadiene at the 1- or 1,4-position led to significantly higher quantum yields, which is in accordance with previous observations with naphthyl-substituted derivatives [29–30]. Apparently, there is no obvious correlation between the quantum yields of the photoreactions and substitution pattern of the norbornadiene in this series, except for a rough trend that naphthyl-linked derivatives, in particular the ones substituted in the 2-position, have lower quantum yields than the phenyl-linked ones. This lack of correlation may indicate that several different factors and processes contribute to different extent to the course of the photoreaction, for example, steric hindrance and resulting torsion angles between the arene unit and norbornadiene, deactivation of the excited state by intersystem crossing (ISC), internal conversion (IC), or quenching by structural relaxation, etc.

**Table 1:** Photochemical and physicochemical parameters of the norbornadiene–quadricyclane interconversion of **1h**–**l**,**n** in cyclohexane.

	λ_max_ [nm]^a^	λ_onset_ [nm]^b^	λ_onset_ [nm]^c^	Φ [%]^d^	Δ*G*^≠^ [kJ mol^−1^]^e^	*t*_1/2_ [h]^f^

**1h**	326	386	366	31 (31)	97	3.6
**1i**	328	352	357	6 (6)	96	3.0
**1j**	332	400	392	10 (5)	97	3.1
**1k**	310	345	336	59 (30)	99	7.8
**1l**	315	345	355	16 (8)	99	7.6
**1n**	345	420	412	1 (0.5)	n.d.^g^	n.d.^g^

^a^Long-wavelength absorption maximum of norbornadiene. ^b^Zero-onset of norbornadiene absorption (at ε = 10 L mol^−1^ cm^−1^). ^c^Long-wavelength absorption of quadricyclane (at ε = 10 L mol^−1^ cm^−1^). ^d^Quantum yield of photoisomerization per photoconversion event, absolute value in brackets, determined by actinometry. ^e^Activation enthalpy of thermally induced cycloreversion of the quadricyclane; determined from Eyring equation. ^f^Half-life of the quadricyclane at 20 °C, determined from Eyring equation. ^g^Not determined.

Finally, the half-life, *t*_1/2_, of the quadricyclane derivatives **2h**–**l**,**n** was determined by kinetic analysis of the thermally induced cycloreversion to the corresponding norbornadiene derivatives **1h**–**l**,**n** at 60 °C. From these data, the rate constants of the cycloreversion reactions were generated according to first-order kinetics and used to calculate the enthalpy and entropy of activation with the Eyring equation (Table 1, cf. Supporting Information File 1, equation S5). The analyses revealed half-lives of the quadricyclanes from 3.0 h to 7.8 h, which are essentially in the same range as the ones of analogues with an acceptor-substituent in the 3-position of the norbornadiene [34]. Notably, the quadricyclane derivatives with sterically more demanding substituents in the 2-position of the norbornadiene unit, such as **1e**, showed longer half-lives. Therefore, compounds **1h**–**l**,**n** have significanty lower half-lives than the corresponding analogues without an alkyne linker [29–30].

## Conclusion

In summary, a small series of mono-, bis-, and tris-norbornadiene derivatives with alkynylbenzene and alkynylnaphthalene core units was made available in reasonable yields by Sonogashira–Hagihara coupling reactions and the potential of these compounds for MOST applications was assessed. In particular, the compounds showed red-shifted absorption of up to 420 nm. Upon direct irradiation, the norbornadienes are converted into the corresponding quadricyclanes, which have short half-lives of only a few hours. The photoisomerization quantum yields were, with one exception, relatively low, as compared with the ones of other, structurally resembling norbornadiene derivatives [30,34]. Moreover, upon prolonged reaction time, significant amounts of unidentified by-products formed. Nevertheless, it was shown exemplarily that the photoisomerization can be performed under milder conditions and upon excitation with visible light in the presence of [Ru(phen)_3_](PF_6_)_2_ as a photosensitizer. Under these conditions, the derivatives **1i** and **1l** were converted quantitatively into quadricyclanes by irradiation with green light. Thus, it was shown with the latter results that the photosensitization with suitable triplet sensitizers enables mild conversion of visible light in chemical energy in quantitative photoreactions of norbornadienes, even in cases that are rather problematic under direct irradiation.

The original target design, starting from the promising class of solely hydrocarbon-based, monoaryl-substituted norbornadienes, aimed primarily at the optimization of the absorption properties without adversely affecting the other advantageous MOST properties. However, although the integration of alkyne-linker units into otherwise unsubstituted arene-linked mono-, bis-, and tris-norbornadienes resulted in the anticipated red shift of the absorption, this slight advantage was established to the disadvantage of other relevant MOST properties, especially photostability. From these results, it may be concluded that the sole addition of alkyne units to optimize MOST materials should be handled carefully because in some cases, like the one reported herein, it may result in an unfavorable tradeoff between the distinct MOST properties. After all, it still remains a challenge to design MOST materials with well-balanced advantageous properties. Based on the results presented herein, it may be proposed that the optimization of monoaryl-substituted norbornadienes along these lines should focus more on the factors that lead to improved stability, energy storage capacity and back reaction of the quadricyclane, and not primarily on the implementation of red-shifted absorption into the visible range. Specifically, the latter can easily be addressed in a more efficient way by the application of a suitable photosensitizer, as shown exemplarily in this work.

## Supporting Information

File 1 Experimental section and complete set of NMR spectra.

## Data Availability

All data that supports the findings of this study is available in the published article and/or the supporting information of this article.
