# Supplementary material for: Extension of the π-system of monoaryl-substituted norbornadienes with acetylene bridges: influence on the photochemical conversion and storage of light energy
Source: Beilstein J Org Chem. 2024 Nov 21;20:3061–8. doi: 10.3762/bjoc.20.254 (PMC11590010; doi:10.3762/bjoc.20.254)
Supplement: File 1 — Experimental section and complete set of NMR spectra. [file Beilstein_J_Org_Chem-20-3061-s001.pdf]

BEILSTEIN JOURNAL OF ORGANIC CHEMISTRY

Supporting Information

for

Extension of the π-system of monoaryl-substituted norbornadienes with acetylene bridges: influence on the photochemical conversion and storage of light energy

Robin Schulte, Dustin Schade, Thomas Paululat, Till J. B. Zähringer, Christoph Kerzig and Heiko Ihmels

Beilstein J. Org. Chem. **2024,** *20,* 3061–3068. doi:10.3762/bjoc.20.254

Experimental section and complete set of NMR spectra

License and Terms: This is a supporting information file under the terms of the Creative Commons Attribution License (https://creativecommons.org/licenses/by/4.0). Please note that the reuse, redistribution and reproduction in particular requires that the author(s) and source are credited and that individual graphics may be subject to special legal provisions.
The license is subject to the *Beilstein Journal of Organic Chemistry* terms and conditions: (https://www.beilstein-journals.org/bjoc/terms)

Table of contents

S1 Equipment S2

S2 Methods S2

S3 Material S3

S4 Synthesis S3

S5 Absorption properties of norbornadiene derivatives **1h**–**l**, **n** S8

S6 Photoreactions and photoisomerization quantum yields S9

S7 Quenching experiments of $[Ru(phen)_3](PF_6)_2$ with **1i** S11

S8 Kinetic studies of the thermal back reaction S12

S9 NMR spectra S14

S9.1 Structure elucidation S14

S10 References S34

S1 Equipment

The NMR spectra were recorded on a JEOL ECZ 500 spectrometer (^{1}H: 500 MHz, ^{13}C: 125 MHz, 25 °C) or on a Varian VNMR-S 600 (^{1}H: 600 MHz, ^{13}C: 150 MHz, 25 °C). In-situ irradiation inside the NMR spectrometer was done with a self-made irradiation setup in a Varian VNMR-S 600.[1] The spectra were referenced to tetramethylsilane in $CDCl_3$ [δ(^{1}H) = 0.00 ppm] or residual solvent signals in $CDCl_3$ [δ(^{13}C) = 77.2 ppm] or benzene-d_6 [δ(^{1}H) = 7.16 ppm, δ(^{13}C) = 128.0 ppm] and processed with the MestReNova software. The melting points were determined with a Büchi 545 (Büchi, Flawil, CH) and are uncorrected. Elemental analysis data were determined in-house (Organic Chemistry, University of Siegen) on a HEKAtech EUROEA combustion analyzer. The absorption spectra were measured on a Varian Cary 100 Bio absorption spectrometer or on a Analytik Jena SPECORD S spectrometer with Hellma quartz glass cuvettes 115 F-QS (d = 10 mm). If not stated otherwise, the absorption measurements were recorded at T = 20 °C as adjusted with a thermostat. The spectra were processed with the software Origin with the implemented smoothing function "adjacent averaging" with the factor of 10.[2] Photoreactions were performed with Thorlabs LED lamp M310L1, Thorlabs LED M340L5, Conrad LED520 (181862) or a LUMOS 43 from Atlas Photonics.The LP980KS setup from Edinburgh Instruments, equipped with an Nd:YAG laser from Litron (Nano LG 300-10) and an Nd:YAG Quantel (Q-smart 450), was employed for transient absorption and time-resolved emission spectroscopy. The frequency-doubled (532 nm) output served as the excitation source. The laser pulse duration was ≈5 ns and the pulse frequency was 10 Hz. The typical pulse energy used for transient absorption and emission studies was ≈20 mJ. A constant laser pulse energy was used within a series of experiments, which was ensured by power measurements before, after and in-between the experiments. The laser power was measured using a pyroelectric detector with attenuator (QE25LP-S-MB-QED-D0) from Gentec-eo combined with the integra software. Detection of transient absorption spectra occurred on an iCCD camera from Andor. Kinetic traces at selected wavelengths were recorded using a photomultiplier tube. The spectroscopic experiments were performed at 293 K using a cuvette holder that allows temperature control. If not stated otherwise the TA spectra were integrated over 100 ns.

S2 Methods

Reaction mixtures were stirred with a magnetic stirring bar. Reaction temperatures refer to the medium that surrounded the reaction vessel. Solvents were usually removed under reduced pressure at 40 °C with a rotatory evaporator. The room temperature (rt) was approx. 22 °C. Air- and/or water-sensitive reactions were carried out under inert atmosphere with Schlenk equipment. The triplet energy transfer rate constant was determined by a Stern–Volmer analysis. This was achieved by lifetime-based measurements of $[Ru(phen)_3](PF_6)_2$ (τ) with varying concentrations c_i of the quencher and fitted according to the Stern–Volmer equation shown below (Equation S1).

$$\frac{\tau_0}{\tau_i} = k_{EnT} \cdot c_i \cdot \tau_0 + 1 \qquad \text{(S1)}$$

S3 Material

Commercially available reagents were obtained from Carbolution [$PdCl_2(PPh_3)_2$], Chemsolute (NEt_3), Riedel de Haen (CuI), Sigma–Aldrich {[$Ru(phen)_3]Cl_2$, 98%}, and Carl Roth, (NH_4PF_6, 98%). 2-Bromonorbornadiene[3], 1-ethynylnaphthalene[4], 2-ethynylnaphthalene[5], 1,4-diethynylbenzene[6], 1,3-diethynylbenzene[6], 1,3,5-triethynyl-benzene[6], 1,4-diethynylnaphthalene[7], and 2,6-diethynylnaphthalene[8] were synthesized according to the literature protocols. The derivatives **1h**–**n** were stored at −30 °C. Technical-grade solvents were distilled prior to use. Column chromatography was carried out with CHROMABOND Flash RS15 (25 µm, capacity: 12 mg–3.6 g).

S4 Synthesis

General procedure (GP A) for the Pd-catalyzed Sonogashira–Hagihara coupling reactions

Under anaerobic conditions, the acetylenes **3a**–**g** (654 µmol–1.23 mmol) were added to a suspension of 2-bromonorbornadiene (**1g**, 1.24 mmol–7.38 mmol), $PdCl_2(PPh_3)_2$ (65.4 µmol–123 µmol), CuI (65.4 µmol–123 µmol) and NEt_3 (2.0 mL–10 mL) in THF (10 mL–25 mL). The resulting mixture was stirred at rt for 18 h. The solvents were removed under reduced pressure and the remaining crude was purified by column chromatography.

1-(Bicyclo[2.2.1]hepta-2,5-dien-2-ylethynyl)naphthalene (**1h**)

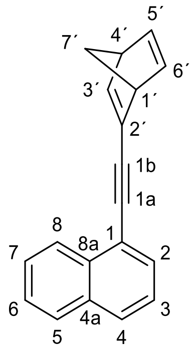

According to GP A, a suspension of 1-ethynylnaphthalene (**3a**, 126 mg, 826 µmol), 2-bromonorbornadiene (**1g**, 212 mg, 1.24 mmol), $PdCl_2(PPh_3)_2$ (57.9 mg, 82.6 µmol), CuI (15.7 mg, 82.6 µmol), and NEt_3 (2.0 mL) in THF (10 mL) was stirred at rt for 18 h. The crude product was purified by column chromatography (*n*-hexane/EtOAc, 99/1, *v*/*v*, R_f = 0.40) to give **1h** as a yellow oil (120 mg, 496 µmol, 60%). – ^{1}H NMR (500 MHz, $CDCl_3$): δ = 2.14 (dt, 1H, ^2J = 6.3 Hz, ^3J = 1.5 Hz, 7´-H), 2.24 (dt, 1H, ^2J = 6.3 Hz, ^3J = 1.5 Hz, 7´-H), 3.73–3.76 (m, 1H, 4´-H), 3.78–3.80 (m, 1H, 1´-H), 6.79 (dd, 1H, ^3J = 4.8 Hz, ^3J = 3.0 Hz, 5´-H), 6.96 (dd, 1H, ^3J = 4.8 Hz, ^3J = 3.0 Hz, 6´-H), 7.11 (d, 1H, ^3J = 3.1 Hz, 3´-H), 7.41 (dd, 1H, ^3J = 8.2 Hz, ^3J = 7.1 Hz, 3-H), 7.50–7.53 (m, 1H, 6-H), 7.55–7.58 (m, 1H, 7-H), 7.65 (dd, 1H, ^3J = 7.2 Hz, ^4J = 1.2 Hz, 2-H), 7.79 (d, 1H, ^3J = 8.2 Hz, 4-H), 7.83–7.85 (m, 1H, 5-H), 8.32–8.34 (m, 1H, 8-H). – ^{13}C NMR (125 MHz, $CDCl_3$): δ = 51.3 (C4´), 56.1 (C1´), 73.7 (C7´), 91.2 (C1b), 95.6 (C1a), 121.5 (C4a), 125.4 (C3), 126.4 (C6), 126.5 (C8), 126.7 (C7), 128.3 (C5), 128.6 (C4), 130.0 (C2), 133.1 (C8a), 133.6 (C1), 138.4 (C2´), 142.6 (C5´), 143.0 (C6´), 148.5 (C3´). – El. Anal. for $C_{19}H_{14}$: calc. (%): C 94.18, H 5.82. found (%): C 93.56, H 6.03.

2-(Bicyclo[2.2.1]hepta-2,5-dien-2-ylethynyl)naphthalene (**1i**)

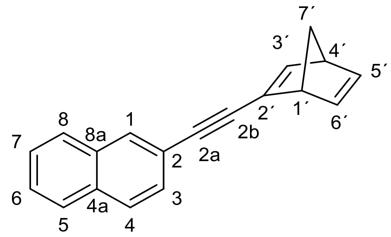

According to GP A, a suspension of 2-ethynylnaphthalene (**3b**, 126 mg, 826 µmol), 2-bromonorbornadiene (**1g**, 212 mg, 1.24 mmol), $PdCl_2(PPh_3)_2$ (57.9 mg, 82.6 µmol), CuI (15.7 mg, 82.6 µmol), and NEt_3 (2.0 mL) in THF (10 mL) was stirred at rt for 18 h. The crude product was purified by column chromatography (*n*-hexane/EtOAc, 99/1, *v*/*v*, R_f = 0.35) to give **1i** as colorless amorphous solid (136 mg, 562 µmol, 68%); mp 79–81 °C. – ^{1}H NMR (500 MHz, $CDCl_3$): δ = 2.11 (dt, 1H, ^2J = 6.3 Hz, ^3J = 1.5 Hz, 7´-H), 2.19 (dt, 1H, ^2J = 6.3 Hz, ^3J = 1.5 Hz, 7´-H), 3.70–3.73 (m, 2H, 1´-H, 4´-H), 6.76–6.78 (m, 1H, 5´-H), 6.92–6.94 (m, 1H, 6´-H), 7.05–7.06 (m, 1H, 3´-H), 7.45–7.50 (m, 3H, 3-H, 6-H, 7-H), 7.76–7.81 (m, 3H, 4-H, 5-H, 8-H), 7.94–7.96 (m, 1H, 1-H). – ^{13}C NMR (125 MHz, $CDCl_3$): δ = 51.3 (C4´), 55.9 (C1´), 73.7 (C7´), 86.6 (C2b), 97.9 (C2a), 121.1 (C2), 126.6 (C6), 126.7 (C7), 127.8 (C5), 127.9 (C8), 128.0 (C4), 128.4 (C3), 131.0 (C1), 132.8 (C4a), 133.1 (C8a), 138.4 (C2´), 142.6 (C5´), 142.9 (C6´), 149.6 (C3´). – El. Anal. for $C_{19}H_{14}$: calc. (%): C 94.18, H 5.82. found (%): C 94.17, H 5.95.

1,4-Bis(bicyclo[2.2.1]hepta-2,5-dien-2-ylethynyl)benzene (**1j**)

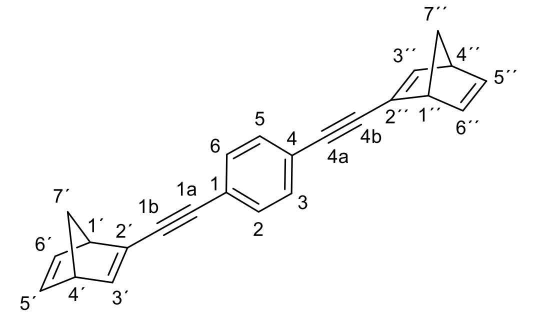

According to GP A, a suspension of 1,4-diethynylbenzene (**3c**, 82.4 mg, 654 µmol), 2-bromonorbornadiene (**1g**, 392 mg, 2.29 mmol), $PdCl_2(PPh_3)_2$ (45.9 mg, 65.4 µmol), CuI (12.4 mg, 65.4 µmol), and NEt_3 (2.0 mL) in THF (10 mL) was stirred at rt for 18 h. The crude product was purified by column chromatography (*n*-hexane, R_f = 0.19) to give **1j** as a yellow amorphous solid (55.0 mg, 180 µmol, 28%); mp 155–160 °C (dec.). – ^{1}H NMR (500 MHz, $CDCl_3$): δ = 1.91 (dt, 2H, ^2J = 6.2 Hz, ^3J = 1.6 Hz, 7´-H, 7´´-H), 2.01 (dt, 2H, ^2J = 6.2 Hz, ^3J = 1.6 Hz, 7´-H, 7´´-H), 3.24–3.31 (m, 2H, 4´-H, 4´´-H), 3.57–3.62 (m, 2H, 1´-H, 1´´-H), 6.49 (dd, 2H, ^3J = 4.9 Hz, ^3J = 3.0 Hz, 5´-H, 5´´-H), 6.71 (dd, 2H, ^3J = 4.9 Hz, ^3J = 2.7 Hz, 6´-H, 6´´-H), 6.88 (d, 2H, ^3J = 3.2 Hz), 7.26 (s, 4H, 2-H, 3-H, 5-H, 6-H). – ^{13}C NMR (125 MHz, $CDCl_3$): δ = 51.5 (C4´, C4´´), 56.2 (C1´, C1´´), 73.6 (C7´, C7´´), 88.7 (C1b, C4b), 97.9 (C1a, C4a), 123.9 (C1, C4), 131.5 (C2, C3, C5, C6), 138.4 (C2´, C2´´), 142.5 (C5´, C5´´), 142.9 (C6´, C6´´), 148.7 (C3´, C3´´). – EI. Anal. for $C_{24}H_{18}$ x 0.5 H_2O: calc. (%): C 91.39, H 6.07. found (%): C 91.15, H 5.84.

1,3-Bis(bicyclo[2.2.1]hepta-2,5-dien-2-ylethynyl)benzene (**1k**)

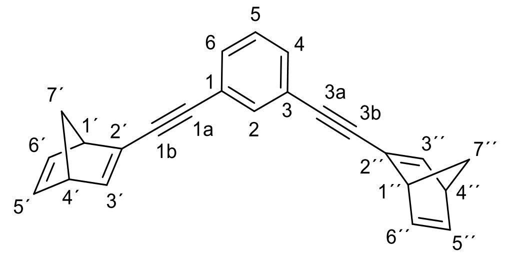

According to GP A, a suspension of 1,3-diethynylbenzene (**3d**, 82.4 mg, 654 µmol), 2-bromonorbornadiene (**1g**, 392 mg, 2.29 mmol), $PdCl_2(PPh_3)_2$ (45.9 mg, 65.4 µmol), CuI (12.4 mg, 65.4 µmol), and NEt_3 (2.0 mL) in THF (10 mL) was stirred at rt for 18 h. The crude product was purified by column chromatography (*n*-hexane/EtOAc, 99/1, *v*/*v*, R_f = 0.31) to give **1k** as a yellow amorphous solid (51.0 mg, 167 µmol, 26%); mp 85–89 °C (dec.). – ^{1}H NMR (500 MHz, $CDCl_3$): δ = 1.90 (dt, 2H, ^2J = 6.2 Hz, ^3J = 1.5 Hz, 7´-H, 7´´-H), 2.01 (dt, 2H, ^2J = 6.2 Hz, ^3J = 1.5 Hz, 7´-H, 7´´-H), 3.24–3.28 (m, 2H, 4´-H, 4´´-H), 3.57–3.59 (m, 2H, 1´-H, 1´´-H), 6.49 (dd, 2H ^3J = 5.1 Hz, ^3J = 3.1 Hz, 5´-H, 5´´-H), 6.71 (dd, 2H ^3J = 5.1 Hz, ^3J = 3.0 Hz, 6´-H, 6´´-H), 6.77 (t, 1H, ^3J = 7.7 Hz, 5-H), 6.87 (d, 2H, ^3J = 3.1 Hz, 3´-H, 3´´-H), 7.25 (dd, 2H, ^3J = 7.7 Hz, ^4J = 1.7 Hz, 4-H, 6-H), 7.72 (t, 1H, ^4J = 1.7 Hz, 2-H). – ^{13}C NMR (125 MHz, $CDCl_3$): δ = 51.4 (C4´, C4´´), 56.1 (C1´, C1´´), 73.6 (C7´, C7´´), 87.6 (C1b, C3b), 97.3 (C1a, C3a), 124.8 (C1, C3), 128.8 (C5), 130.8 (C4, C6), 134.3 (C2), 138.6 (C2´, C2´´), 142.4 (C5´, C5´´), 142.9 (C6´, C6´´), 148.7 (C3´, C3´´). – EI. Anal. for $C_{24}H_{18}$: calc. (%): C 94.08, H 5.92. found (%): C 94.06, H 5.98.

1,3,5-Tris(bicyclo[2.2.1]hepta-2,5-dien-2-ylethynyl)benzene (**1l**)

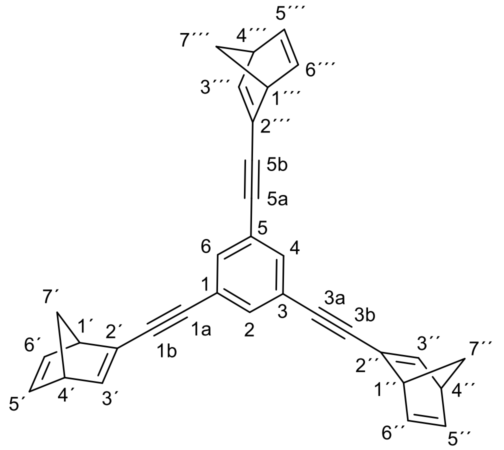

According to GP A, a suspension of 1,3,5-triethynylbenzene (**3e**, 185 mg, 1.23 mmol), 2-bromonorbornadiene (**1g**, 1.26 g, 7.38 mmol), $PdCl_2(PPh_3)_2$ (288 mg, 410 µmol), CuI (77.9 mg, 410 µmol), and NEt_3 (10 mL) in THF (25 mL) was stirred at rt for 18 h. The crude product was purified by column chromatography (*n*-hexane/EtOAc, 99/1, *v*/*v*, R_f = 0.21) to give **1l** as a colorless amorphous solid (198 mg, 470 µmol, 40%); mp 170–178 °C (dec.). – ^{1}H NMR (500 MHz, $CDCl_3$): δ = 1.90 (dt, 3H, ^2J = 6.2 Hz, ^3J = 1.3 Hz, 7´-H, 7´´-H, 7´´´-H), 1.99 (dt, 3H, ^2J = 6.2 Hz, ^3J = 1.3 Hz, 7´-H, 7´´-H, 7´´´-H), 3.24–3.27 (m, 3H, 4´-H, 4´´-H, 4´´´-H), 3.53–3.56 (m, 3H, 1´-H, 1´´-H, 1´´´-H), 6.49 (dd, 3H, ^3J = 4.9 Hz, ^3J = 3.1 Hz, 5´-H, 5´´-H, 5´´´-H), 6.70 (dd, 3H, ^3J = 4.9 Hz, ^3J = 3.0 Hz, 6´-H, 6´´-H, 6´´´-H), 6.85 (d, 3H, ^3J = 3.2 Hz, 3´-H, 3´´-H, 3´´´-H), 7.53 (s, 3H, 2-H, 4-H, 6-H). – ^{13}C NMR (125 MHz, $CDCl_3$): δ = 51.5 (C4´, C4´´, C4´´´), 56.1 (C1´, C1´´, C1´´´), 73.6 (C7´, C7´´, C7´´´), 88.0 (C1b, C3b, C5b), 96.6 (C1a, C3a, C5a), 125.2 (C1, C3, C5), 133.4 (C2, C4, C6), 138.5 (C3´, C3´´, C3´´´), 142.4 (C5´, C5´´, C5´´´), 142.9 (C6´, C6´´, C6´´´), 149.1 (C3´, C3´´, C3´´´). – El. Anal. for $C_{33}H_{24}$ x 1 H_2O: calc. (%): C 90.38, H 5.38. found (%): C 90.49, H 5.76.

1,4-Bis(bicyclo[2.2.1]hepta-2,5-dien-2-ylethynyl)naphthalene (**1m**)

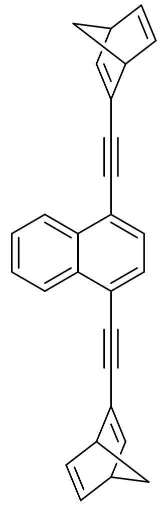

According to GP A, a suspension of 1,4-diethynylnaphthalene (**3f**, 120 mg, 682 µmol), 2-bromonorbornadiene (**1g**, 409 mg, 2.39 mmol), $PdCl_2(PPh_3)_2$ (47.9 mg, 86.2 µmol), CuI (13.0 mg, 86.2 µmol), and NEt_3 (2.0 mL) in THF (10 mL) was stirred at rt for 18 h. Further column chromatography (*n*-hexane/EtOAc, 95/5, *v*/*v*, R_f = 0.51) gave a fraction which accoding to ^{1}H NMR-spectroscopic analysis contained product **1m**, along with byproducts, which could not be further separated.

2,6-Bis(bicyclo[2.2.1]hepta-2,5-dien-2-ylethynyl)naphthalene (**1n**)

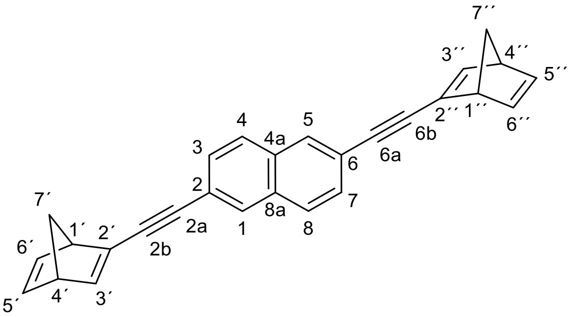

According to GP A, a suspension of 2,6-diethynylnaphthalene (**3g**, 120 mg, 682 µmol), 2-bromonorbornadiene (**1g**, 409 mg, 2.39 mmol), $PdCl_2(PPh_3)_2$ (47.9 mg, 86.2 µmol), CuI (13.0 mg, 86.2 µmol), and NEt_3 (2.0 mL) in THF (10 mL) was stirred at rt for 18 h. The crude product was purified by column chromatography (*n*-hexane/EtOAc, 9/1, *v*/*v*, R_f = 0.64) to give **1n** as a yellow amorphous solid (77.0 mg, 216 µmol, 32%); mp 160–165 °C (dec.). – ^{1}H NMR (500 MHz, $CDCl_3$): δ = 2.11 (dt, 2H, ^2J = 6.3 Hz, ^3J = 1.3 Hz, 7´-H, 7´´-H), 2.19 (dt, 2H, ^2J = 6.3 Hz, ^3J = 1.3 Hz, 7´-H, 7´´-H), 3.70–3.74 (m, 4H, 1´-H, 1´´-H, 4´-H, 4´´-H), 6.75–6.79 (m, 2H, 5´-H, 5´´-H), 6.91–6.95 (m, 2H, 6´-H, 6´´-H), 7.04–7.08 (m, 2H, 3´-H, 3´´-H), 7.47 (dd, 2H, ^3J = 8.5 Hz, ^4J = 1.4 Hz, 3-H, 7-H), 7.69 (d, 2H, ^3J = 8.5 Hz, 4-H, 8-H), 7.87–7.90 (m, 2H, 1-H, 5-H). – ^{13}C NMR (125 MHz, $CDCl_3$): δ = 51.3 (C4´, C4´´), 55.9 (C1´, C1´´), 73.7 (C7´, C7´´), 87.3 (1-C**C**-2´, 6-C**C**-2´´), 97.8 (C2b, C6b), 121.8 (C2a, C6a), 127.8 (C4, C8), 129.0 (C3, C7), 130.7 (C1, C5), 132.4 (C4a, C8a), 138.3 (C2´, C2´´), 142.6 (C5´, C5´´), 142.9 (C6´, C6´´), 149.0 (C3´, C3´´). – El. Anal. for $C_{28}H_{20}$ x H_2O: calc. (%): C 89.81, H 5.91. found (%): C 88.30, H 5.31.

S5 Absorption properties of norbornadiene derivatives 1h–l, n

Starting from stock solutions in cyclohexane (1.0 mM), solutions of **1h–l**, **n** (c = 20 µM, V = 2.00 mL) were prepared in cyclohexane, MeCN, EtOH, $CHCl_3$, EtOAc, and benzene, and the absorption spectrum was recorded (Figure S1). The resulting extinction coefficients were determined according to the Lambert–Beer law and are listed in Table S1.

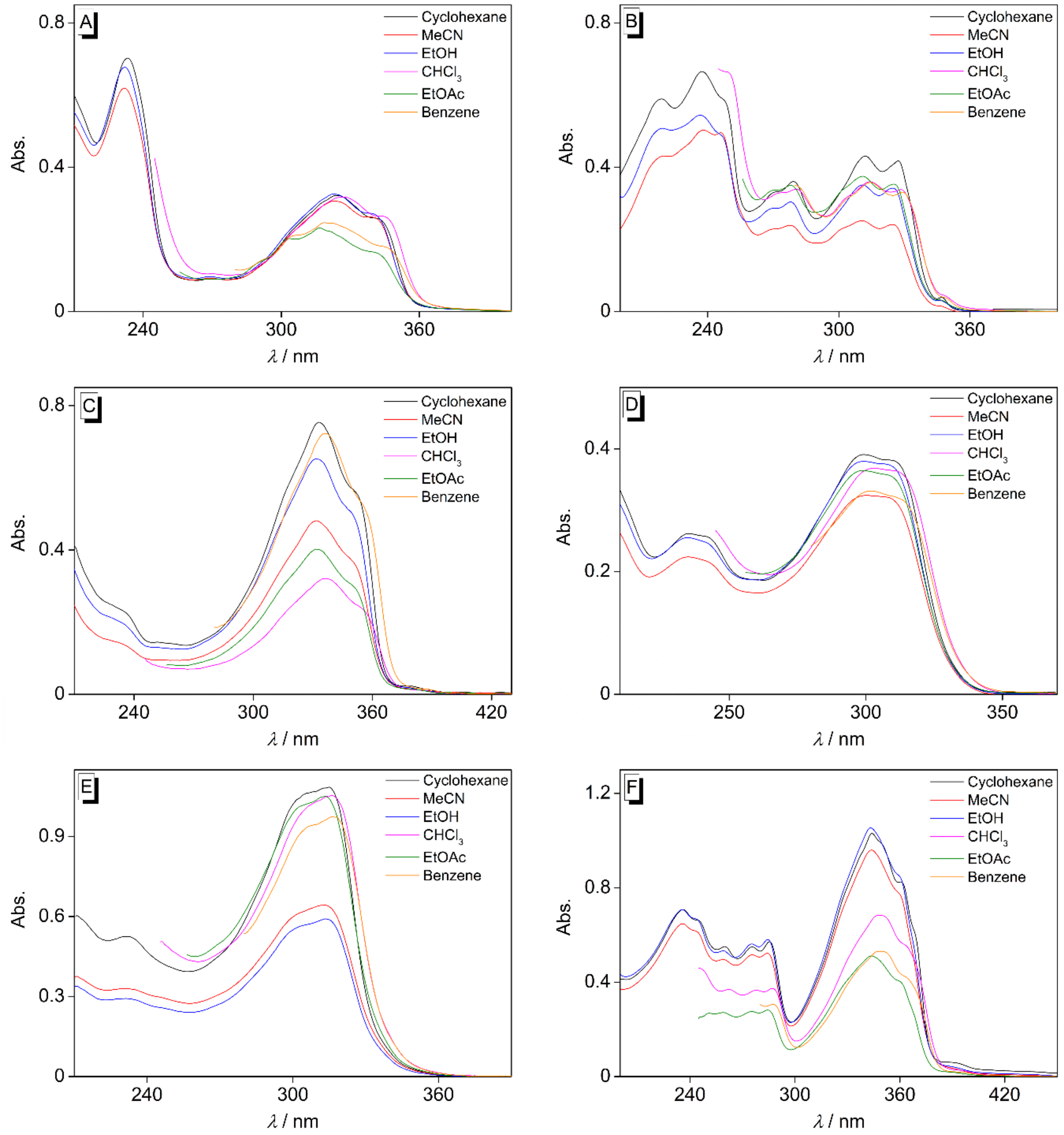

Figure S1: Absorption spectra of **1h** (A), **1i** (B), **1j** (C), **1k** (D), **1l** (E), and **1n** (F) in different solvents (c = 20 µM).

Table S1: Extinction coefficients (in L mol^{-1} cm^{-1}) of **1h–l**, **n** in different solvents.

Solvent	**1h**[a]	**1i**[b]	**1j**[c]	**1k**[d]	**1l**[e]	**1n**[f]
Cyclohexane	16040	20850	37580	19050	54420	51370
MeCN	15190	11950	24250	15930	32000	47880
EtOH	16030	15210	32510	18540	29700	52320
$CHCl_3$	15820	16730	15600	18270	52770	33690
EtOAc	10310	17540	20150	17650	52320	25450
Benzene	11840	20850	37580	19050	48020	26110

[a] Determined at 326 nm. [b] Determined at 328 nm. [c] Determined at 332 nm. [d] Determined at 310 nm. [e] Determined at 315 nm. [f] Determined at 345 nm.

S6 Photoreactions and photoisomerization quantum yields

Samples of the substrates **1h–l**, **n** (c = 20 µM, V = 2.00 mL) were irradiated at λ_{ex} = 315 nm (**1h**, **1i**, **1k**, and **1l**) or at λ_{ex} = 340 nm (**1j**, **1n**), and the photoreaction was monitored by absorption spectroscopy. The reaction was stopped when no further changes in the spectra were detected (Figure 2). In addition, the derivatives **1i** and **1n** (c = 20 µM, V = 2.00 mL) were irradiated in the presence of [Ru(phen)$_3$(PF$_6$)$_2$] (c = 20 µM) at λ_{ex} = 520 nm and the photoreaction was monitored photometrically until no changes in the spectra were occurred. The photoreactions were also followed by ^{1}H NMR spectroscopy. For this purpose, samples of the substrates (c = 3 mM, V = 550 µL) were irradiated with Thorlabs LED M310L1 at λ_{ex} = 310 nm (**1h**, **1i**, **1k**, and **1l**) or with Thorlabs LED M340L5 at λ_{ex} = 340 nm (**1j**, **1n**), followed by ^{1}H NMR spectroscopic analysis (Figures S35, S36, S37, S38, S39, and S40). Because the signals of the corresponding quadricyclanes **2h–l**, **n** could not be clearly identified due to overlaps with decomposition products, no data was generated for **2h–l**, **n**. In addition, **1i** was followed by in-situ irradiation (c = 15 mM, V = 550 µL, λ_{ex} = 520 nm, 10.0 mA per LED) studies in the presence of [Ru(phen)$_3$(PF$_6$)$_2$] (c = 300 µM) in MeCN-d_3 (V = 550 µL) within the NMR spectrometer (Figure S41). For this monitoring of the photoreaction by NMR spectroscopy a self-made NMR setup was used.[1] The irradiation insert of λ_{ex} = 520 nm uses Nichia NCGE17 LEDs (www.leds.de-Article-number 32625). The measured light intensity was 3.2 mW for one LED with 50.0 mA current. The light intensity in the NMR probe towards the NMR active volume was 10.9 mW.

The photoisomerization quantum yields were determined by actinometry with potassium ferrioxalate as reference and irradiation with Thorlabs M310L1 (310 nm) for **1h**, **1i**, **1k**, and **1l** or with Thorlabs LED M340L5 (340 nm) for **1j** and **1n**.[9] First, the photon flux of the irradiation sources was determined according to literature protocol.[10] The values of the light intensity were I_o = 8.53 10^{-8} einstein L^{-1} s^{-1} (M310L1) and I_o = 1.96 10^{-7} einstein L^{-1} s^{-1} (M340L5) (Figure S2).

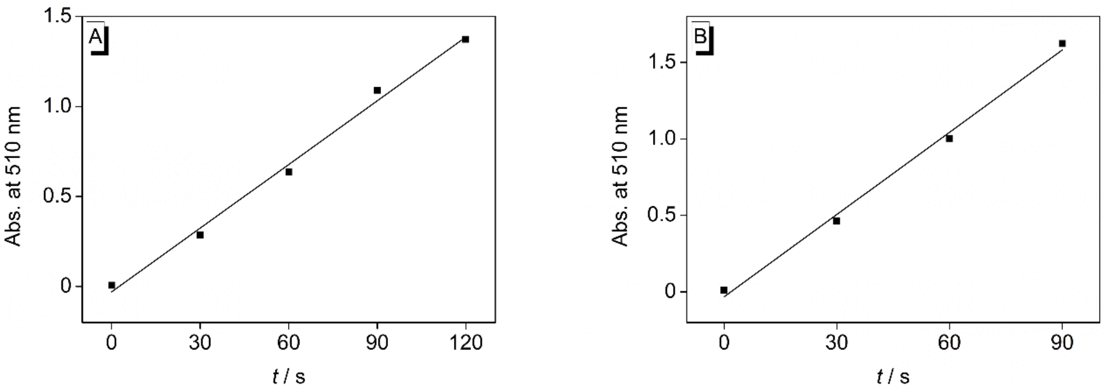

Figure S2: Determination of the photon flux for Thorlabs M10L1 (A) and for Thorlabs M340L5 (B).

The quantum yields of the photoisomerization reactions were determined according to the established procedure (Regime 1: Total absorption regime).[10] The concentrations of the solutions was prepared with Abs. > 2 (c = 60.0–250 µM) at the irradiation wavelength to give a linear dependence between the decrease of absorption and the irradiation time. The quantum yield was determined from the slope of the resulting linear regression (Figure S3, Equation S2).

$$[A] = [A_0] - \frac{\Phi_A\, I}{N_A\, V}\, t_{irr} \qquad \text{(S2)}$$

In Equation S2, $[A]$ is the concentration of norbornadiene, $[A_0]$ is the starting concentration, Φ_A is the quantum yield, I is the photon flux, N_A is the Avogadro number, V the volume and t_{irrad} the irradiation time.

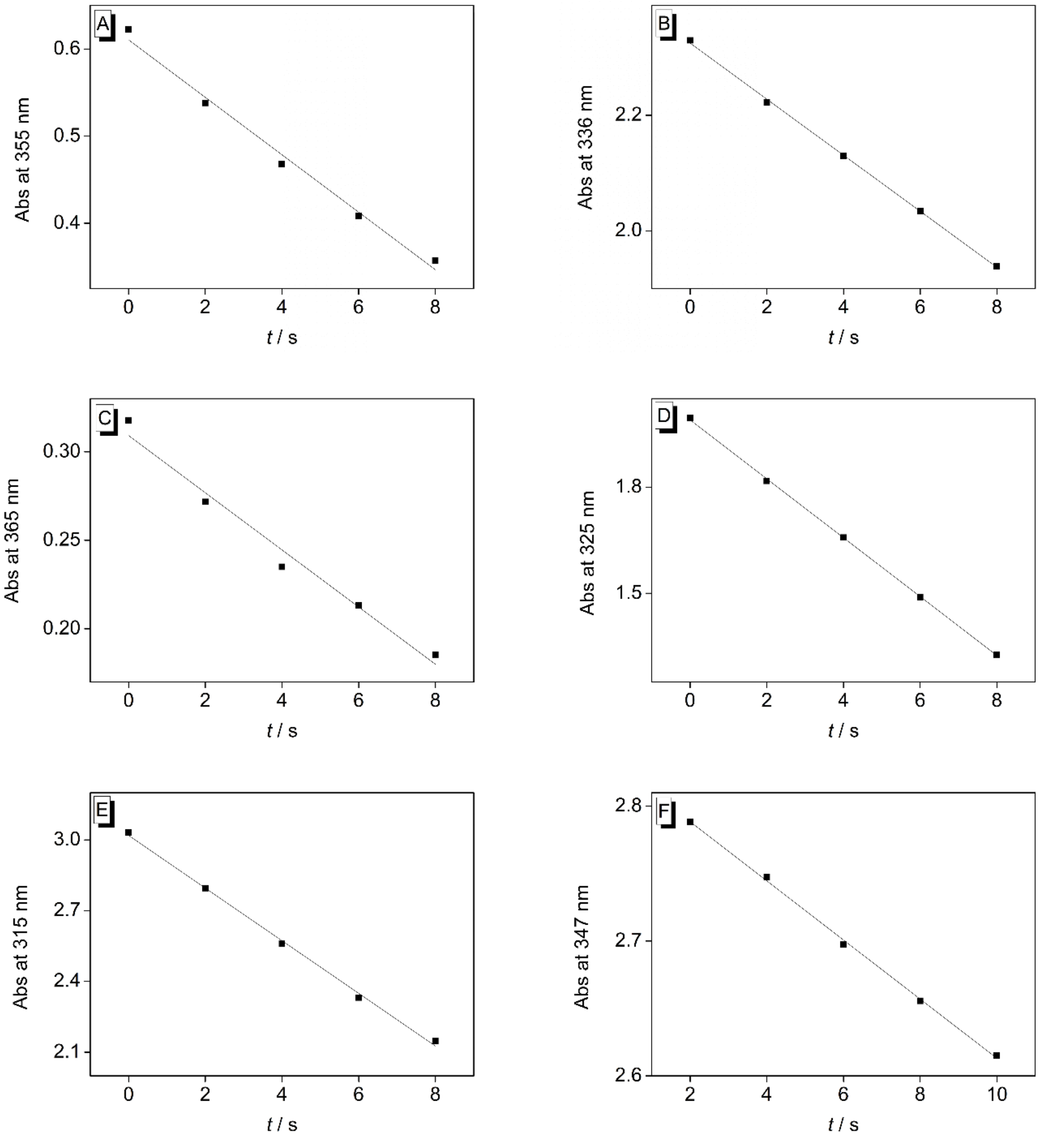

Figure S3: Photometric monitoring of the photoisomerization of **1h** (A), **1i** (B), **1j** (C), **1k** (D), **1l** (E), and **1n** (F) by a plot of the absorption of the substrate versus time. The straight line shows the fit from linear regression analysis, which was used to calculate the quantum yields.

S7 Quenching experiments of $[Ru(phen)_3](PF_6)_2$ with 1i

Previously, we were able to quantitively isomerize naphthyl- and phenyl-substituted norbornadienes by triplet sensitization with visible light.[11] Of the tested compounds, 2-(bicyclo[2.2.1]hepta-2,5-dien-2-yl)naphthalene, is structurally related to **1i** and is lacking the acetylene-bridge (see Figure S4, right). While direct excitation of this photoswitch requires harsh UV light, the triplet energy was determined at 2.41 eV making it accessible by visible-light absorbing photosensitizers. A triplet energy transfer rate constant of $[Ru(phen)_3](PF_6)_2$ with the reference compound is reported as k_{EnT} = 0.83 10^9 M^{-1} s^{-1} [11]. We therefore conducted quenching experiments of triplet-excited $[Ru(phen)_3(PF_6)_2]$ with **1i** as a direct comparison (Figure S4, left). A Stern–Volmer analysis revealed efficient triplet energy transfer to **1i**. Indeed, the energy transfer rate, k_{EnT} = 5.8 10^9 M^{-1} s^{-1}, is roughly higher by one order of magnitude compared to the reference compound. It seems therefore natural to assume that the triplet energy of **1i** is well below 2.4 eV.

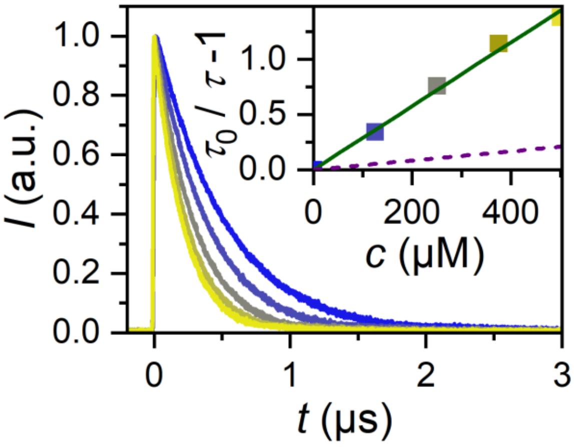

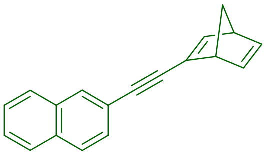

1i

Reference compound:

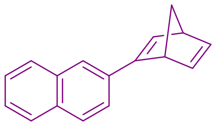

2-(bicyclo[2.2.1]hepta-2,5-dien-2-yl)naphthalene

Figure S4: Left: Emission quenching experiments with detection at 600 nm of 20 µM $[Ru(phen)_3](PF_6)_2$ with varying concentrations of **1i** in Ar-saturated MeCN using 532 nm laser excitation. Inset: Stern–Volmer analysis. The dashed line in the inset indicates the previously measured quenching kinetics for the reference compound. Right: Molecular structures of **1i** and the reference compound 2-(bicyclo[2.2.1]hepta-2,5-dien-2-yl)naphthalene.

Additionally, we recorded transient absorption spectra of a solution containing 20 µM $[Ru(phen)_3](PF_6)_2$ and 500 µM **1i**. The spectra were recorded right after excitation, resembling the typical triplet–triplet absorption spectrum of $[Ru(phen)_3](PF_6)_2$, and after deactivation, where only baseline level is observed. This enables us to exclude electron transfer quenching reactions, which typically yield longer-lived transient photoproducts after quenching[12]. The absence of a triplet-excited **1i** is likely due to a very short triplet lifetime typical for norbornadienes[11,13].

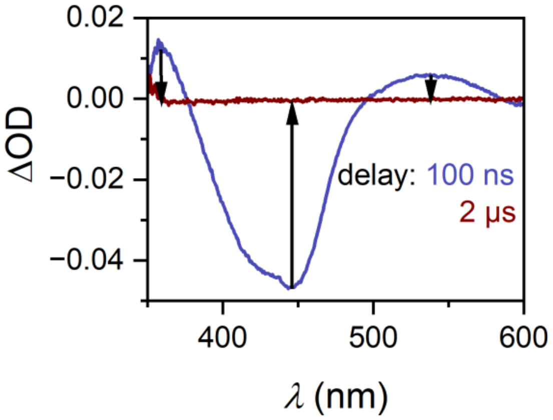

Figure S5: Transient absorption spectrum recorded of an Ar-saturated solution with 20 µM $[Ru(phen)_3](PF_6)_2$ and 500 µM **1i** using 532 nm laser pulses for excitation. The spectra were recorded 100 ns and 2 µs after laser excitation.

S8 Kinetic studies of the thermal back reaction

The thermally induced back reactions from the quadricyclanes to the norbornadiene derivatives were monitored photometrically. Samples of the norbornadienes **1h**–**l**, **n** in cyclohexane (c = 20 µM) were irradiated until no further changes in the absorption could be detected with a LED 310 nm for **1h**, **1i**, **1k**, and **1l** or a LED 340 nm for **1j** and **1n**. The increase of the norbornadiene absorption was determined photometrically at 60 °C (Figure S4).

Considering a unimolecular back reaction to the norbornadiene, the data were analyzed according to first-order kinetics (Equaiton S3) [14].

$$-\frac{\mathrm{d}\,[\mathrm{QC}]}{\mathrm{d}t}=\frac{\mathrm{d}\,[\mathrm{NBD}]}{\mathrm{d}t}=k \tag{S3}$$

In Equation S3, [QC] is the concentration of the quadricyclane, [NBD] is the concentration of the norbornadiene, and k is the rate constant of the thermal back reaction at a certain temperature.

The rate constants k were obtained by an exponential fit (Equation S4). The Eyring equation was used for the calculation of the free activation enthalpy $\Delta G^{\neq}$ of the reaction (Equation S4 and S5)[14].

$$k=\frac{\mathrm{k_B}T}{\mathrm{h}}\,\mathrm{e}^{-\frac{\Delta G^{\neq}}{\mathrm{R}T}} \tag{S4}$$

$$\Delta \mathrm{G}^{\neq}=\mathrm{R}T\left(23.760+\ln\left(\frac{T}{k}\right)\right) \tag{S5}$$

In Equation S4 and S5, k_B is the Boltzmann constant, h is the Planck constant, R is universal gas constant, T is the temperature in K and $\Delta G^{\neq}$ is the free activation enthalpy.

With the obtained values, the half-lifes of the quadricyclanes **2f–j** were calculated at 25 °C with Equation S6. The results are listed in Table 1 in the main text.

$$t_{1/2} = \frac{\ln(2)}{k} \qquad \text{(S6)}$$

In Equation S6, $t_{1/2}$ is the half-life time and k is the rate constant of the thermal back reaction.

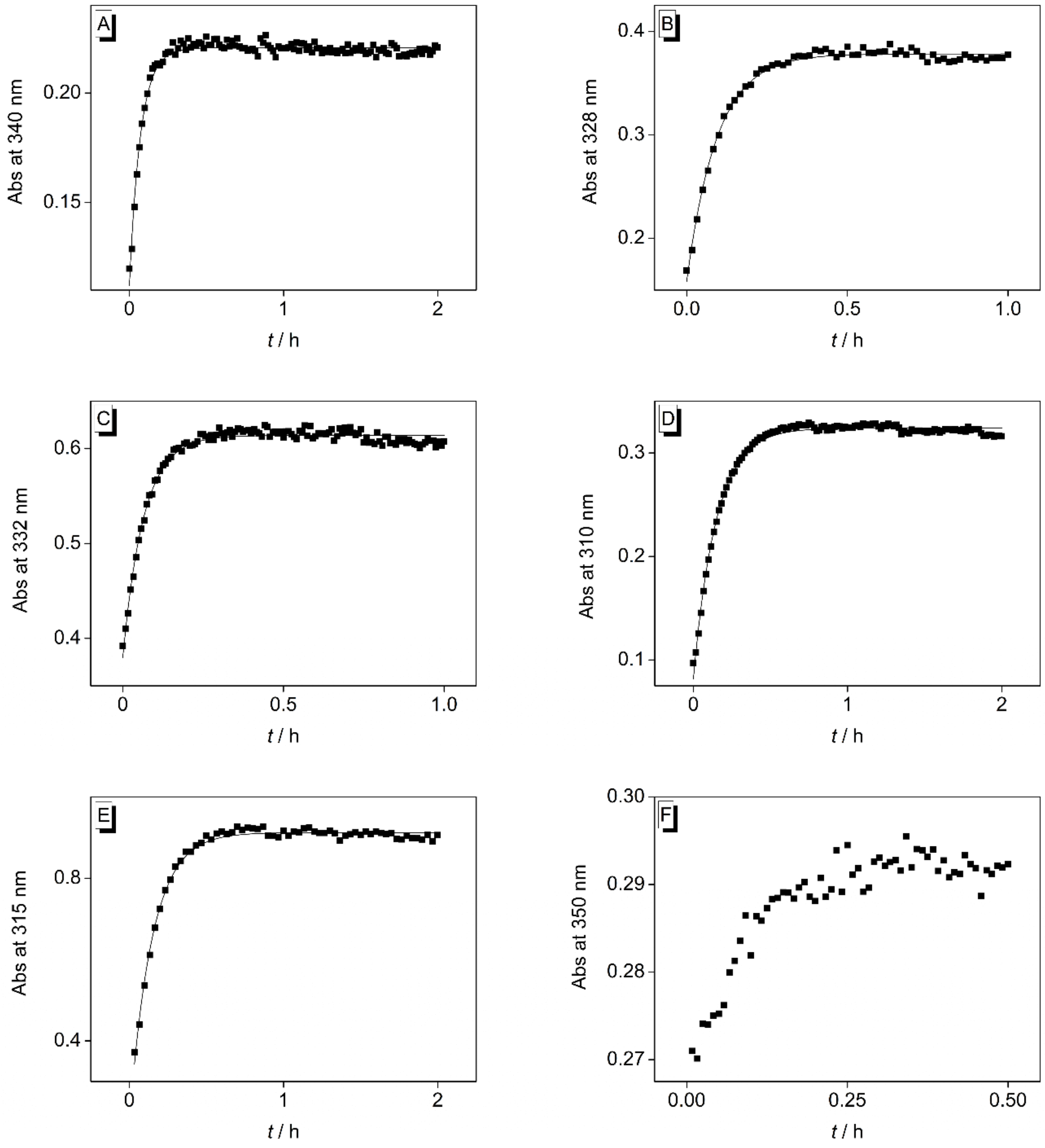

Figure S6: Photometric monitoring of the back conversion of **1h** (A), **1i** (B), **1j** (C), **1k** (D), **1l** (E), and **1n** (F) to the corresponding norbornadienes at 60 °C. The data were fitted to an exponential function (Equation S2) to obtain the rate constants *k* at a particular temperature.

S9 NMR spectra

S9.1 Structure elucidation

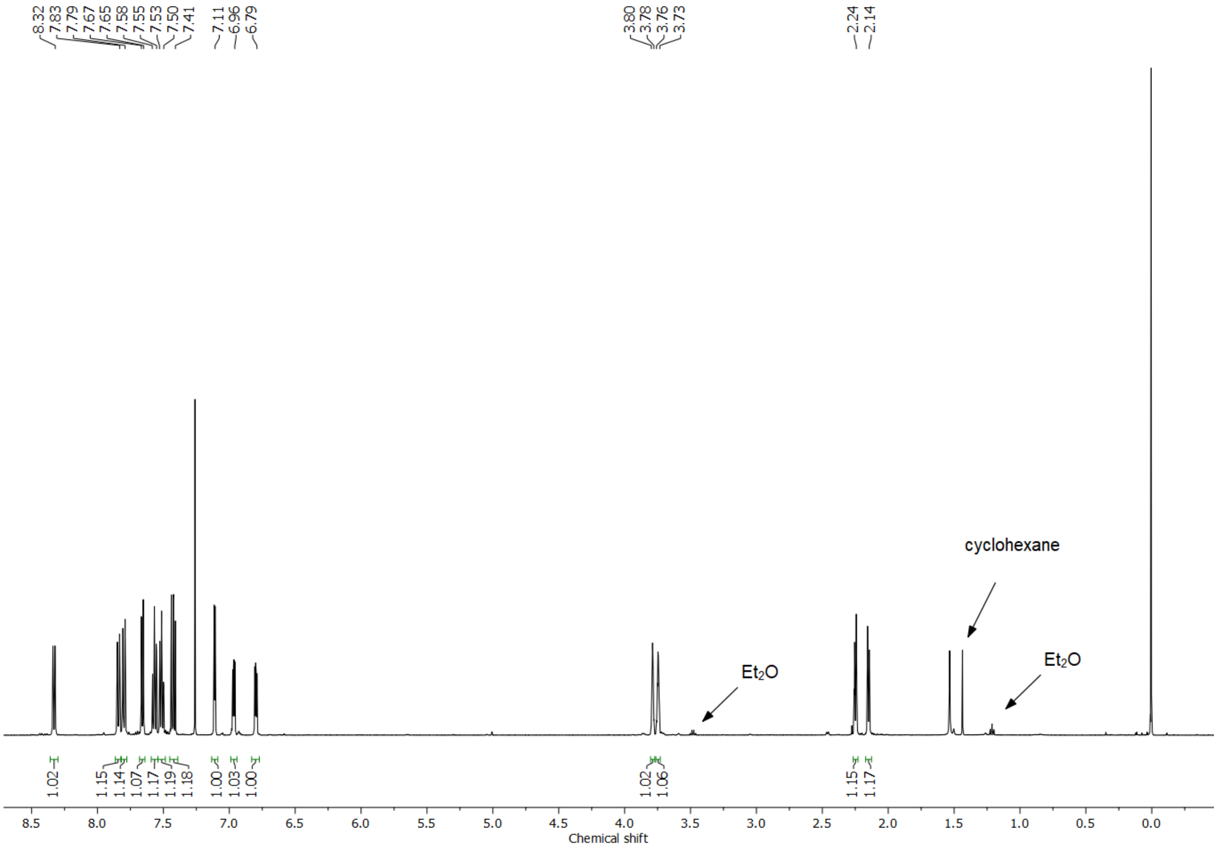

Figure S7: ^{1}H NMR spectrum (500 MHz) of **1h** in $CDCl_3$.

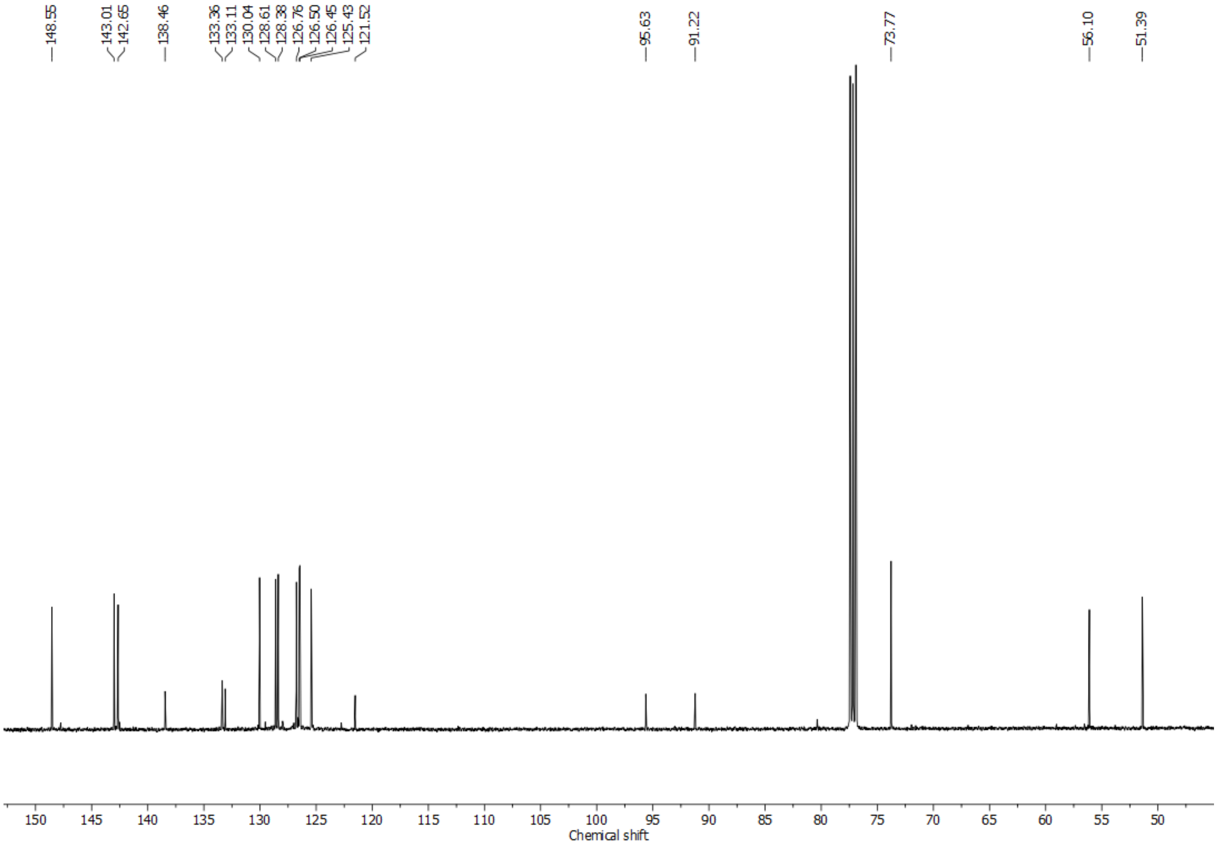

Figure S8: ^{13}C NMR spectrum (125 MHz) of **1h** in $CDCl_3$.

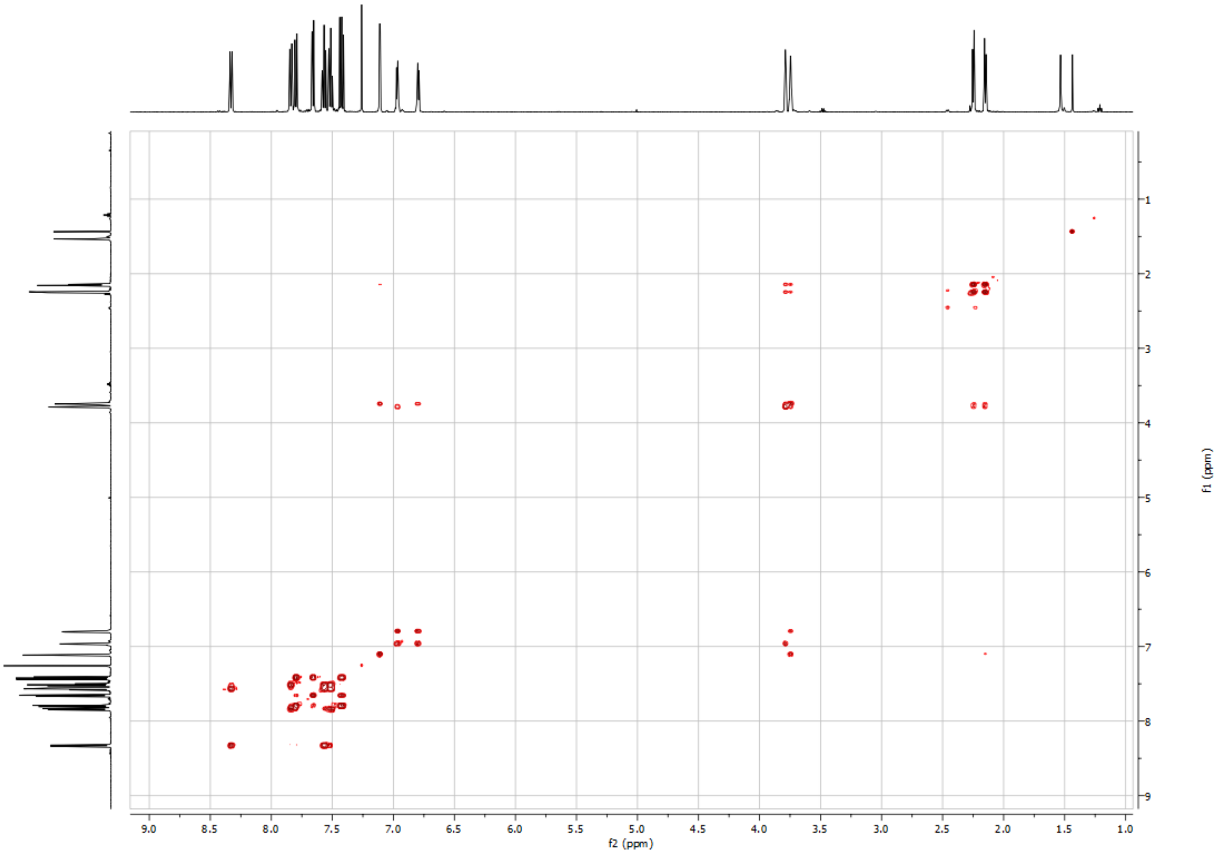

Figure S9: H,H-COSY NMR spectrum (500 MHz) of **1h** in $CDCl_3$.

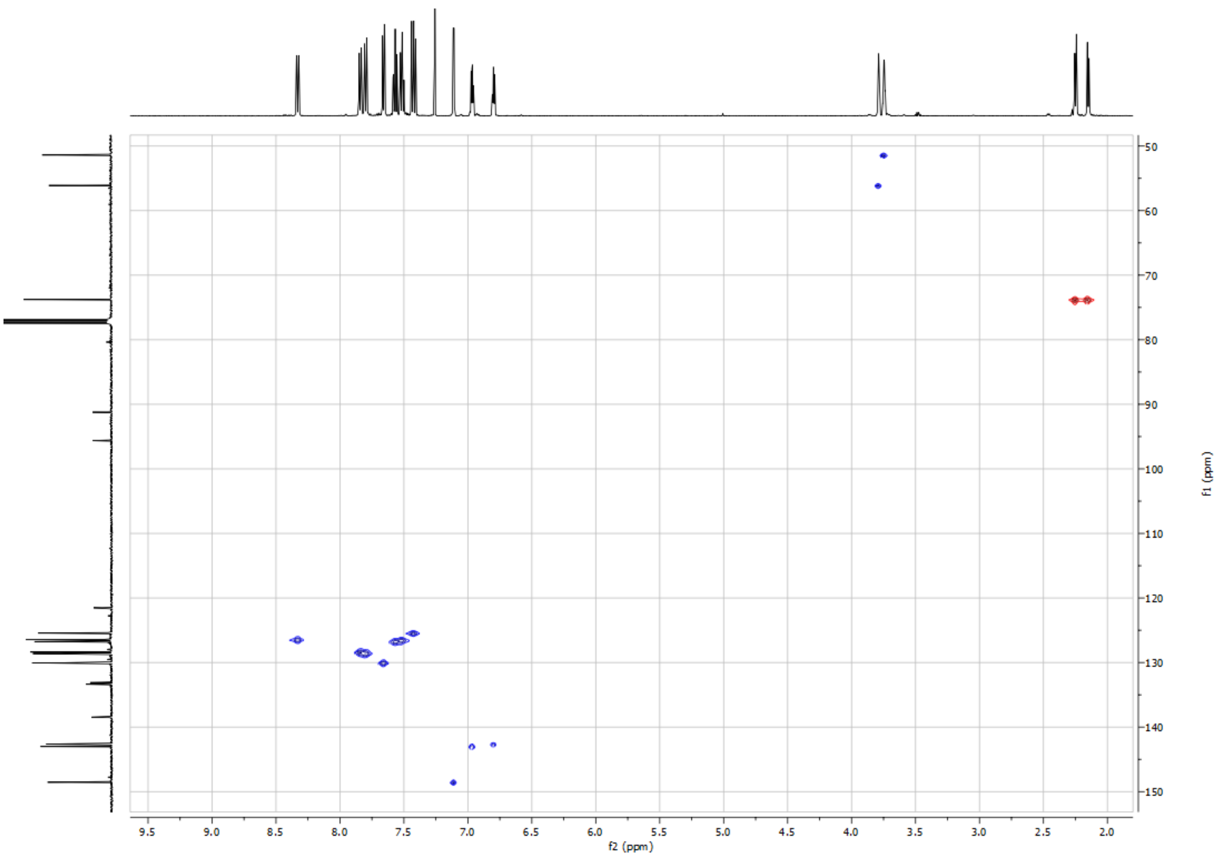

Figure S10: HSQC NMR spectrum (500 MHz) of **1h** in $CDCl_3$.

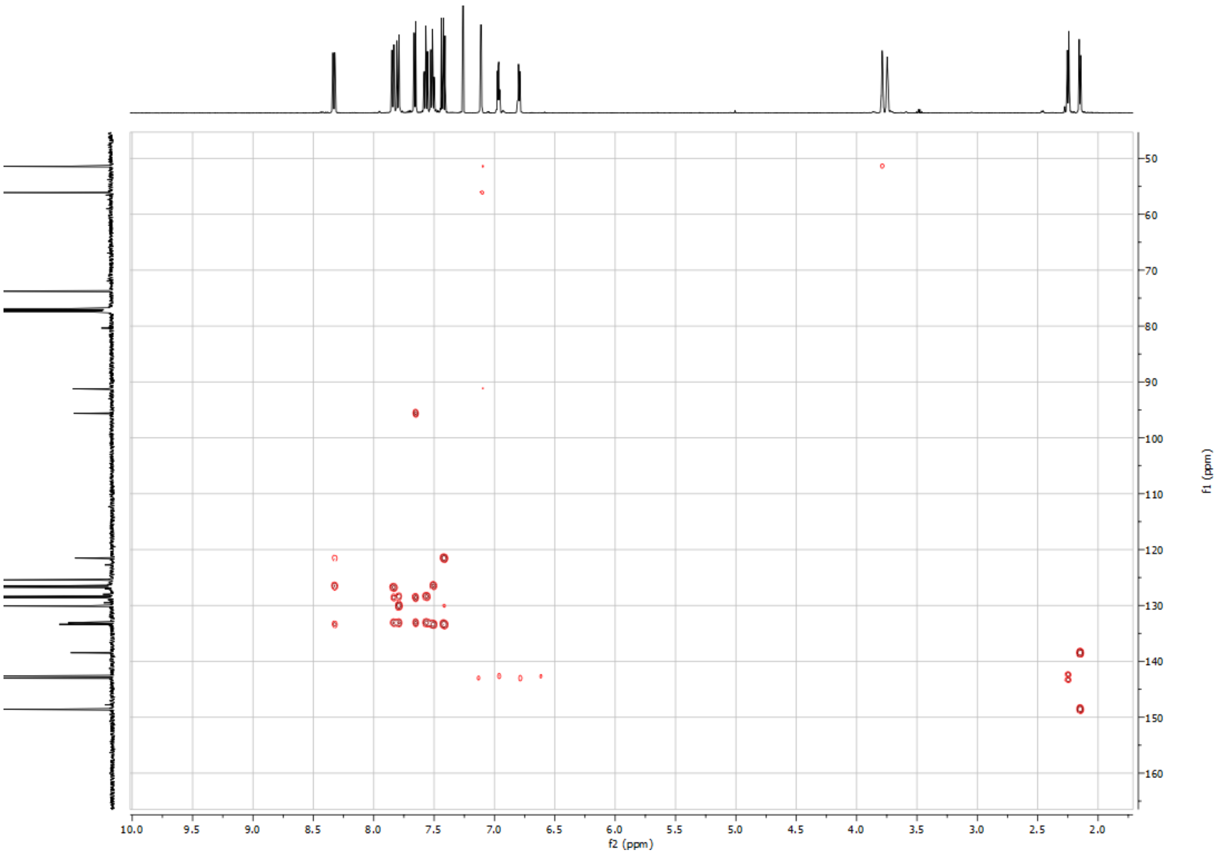

Figure S11: HMBC NMR spectrum (500 MHz) of **1h**in $CDCl_3$.

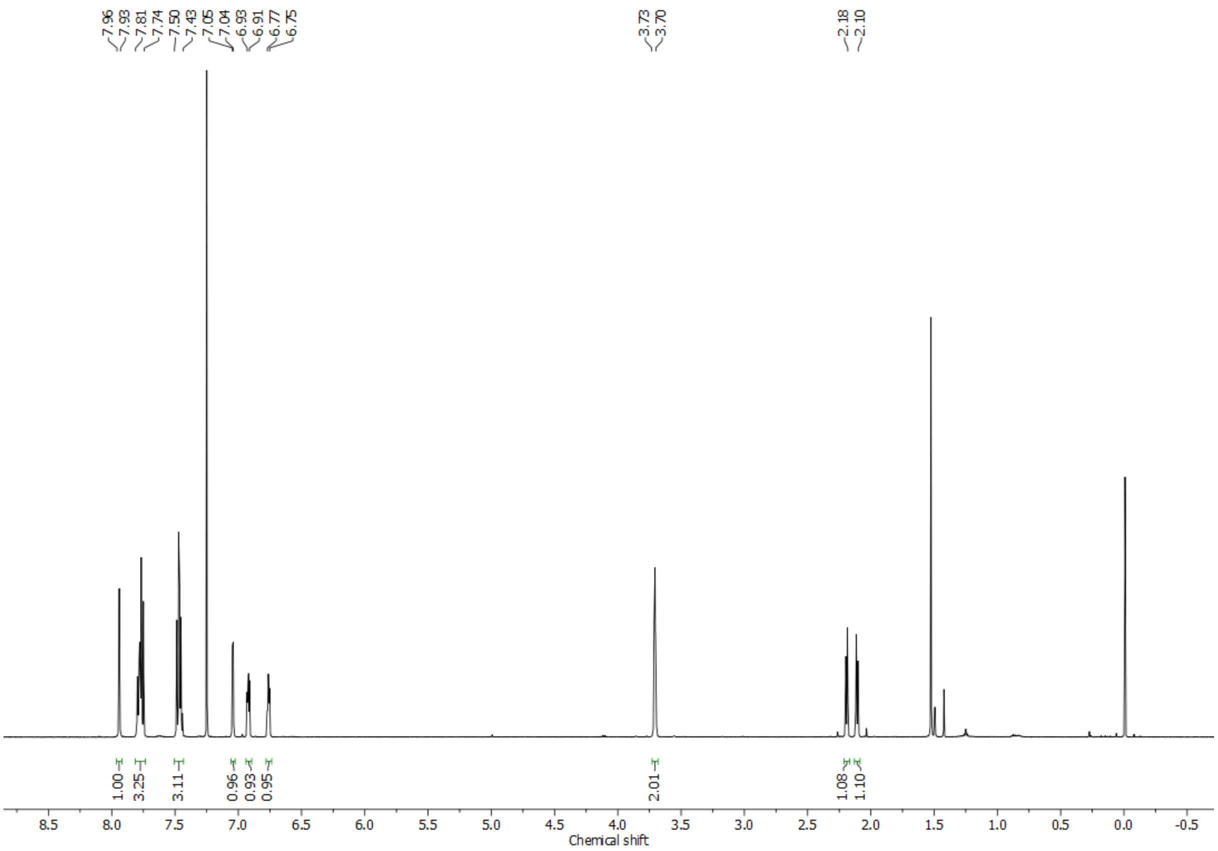

Figure S12: ^{1}H NMR spectrum (500 MHz) of **1i** in $CDCl_3$.

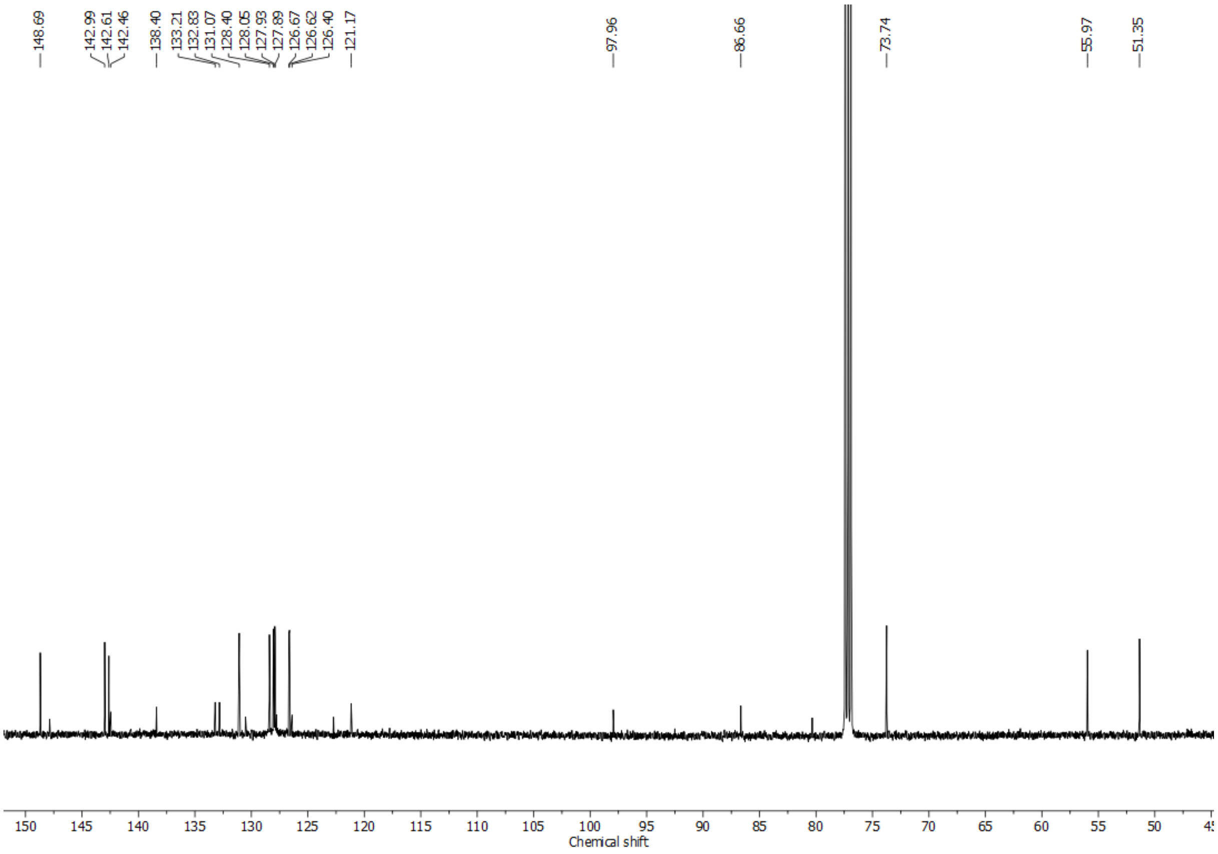

Figure S13: ^{13}C NMR spectrum (125 MHz) of **1i** in $CDCl_3$.

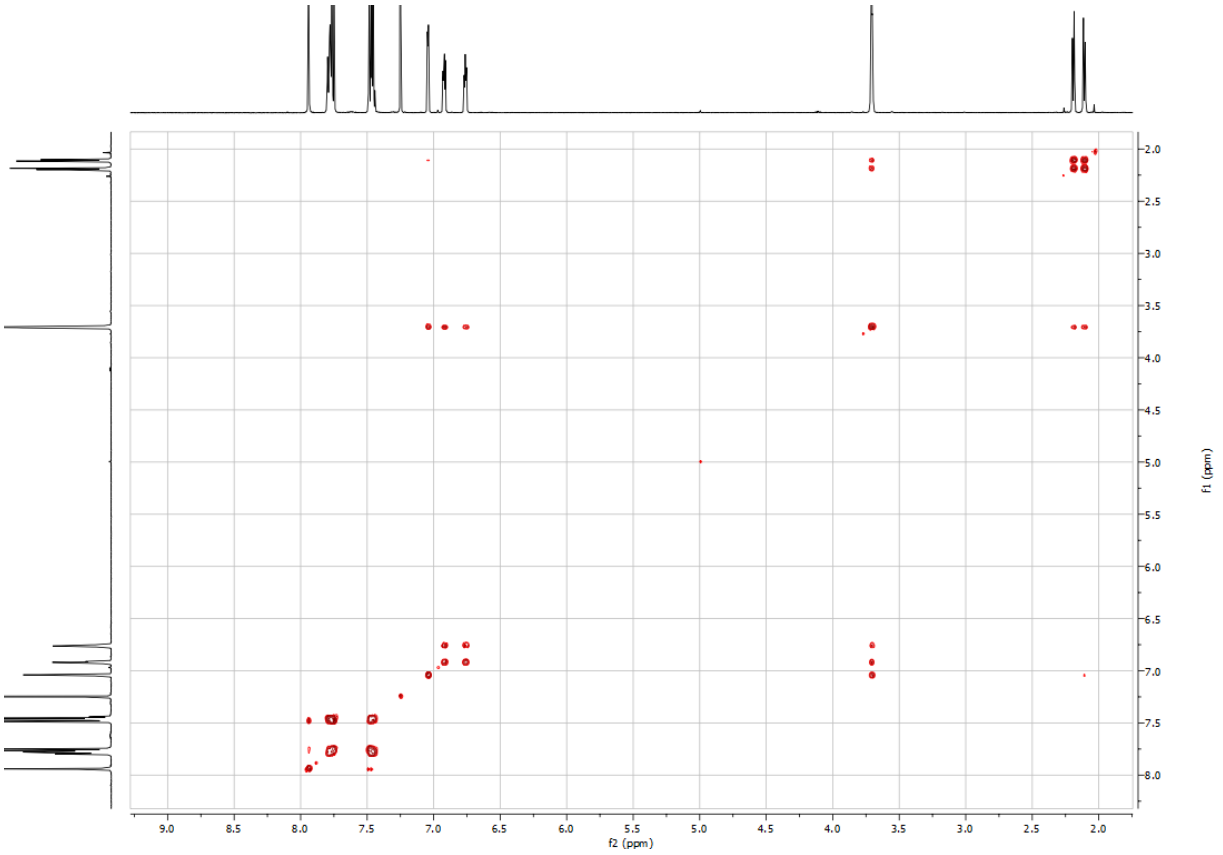

Figure S14: H,H-COSY NMR spectrum (500 MHz) of **1i** in $CDCl_3$.

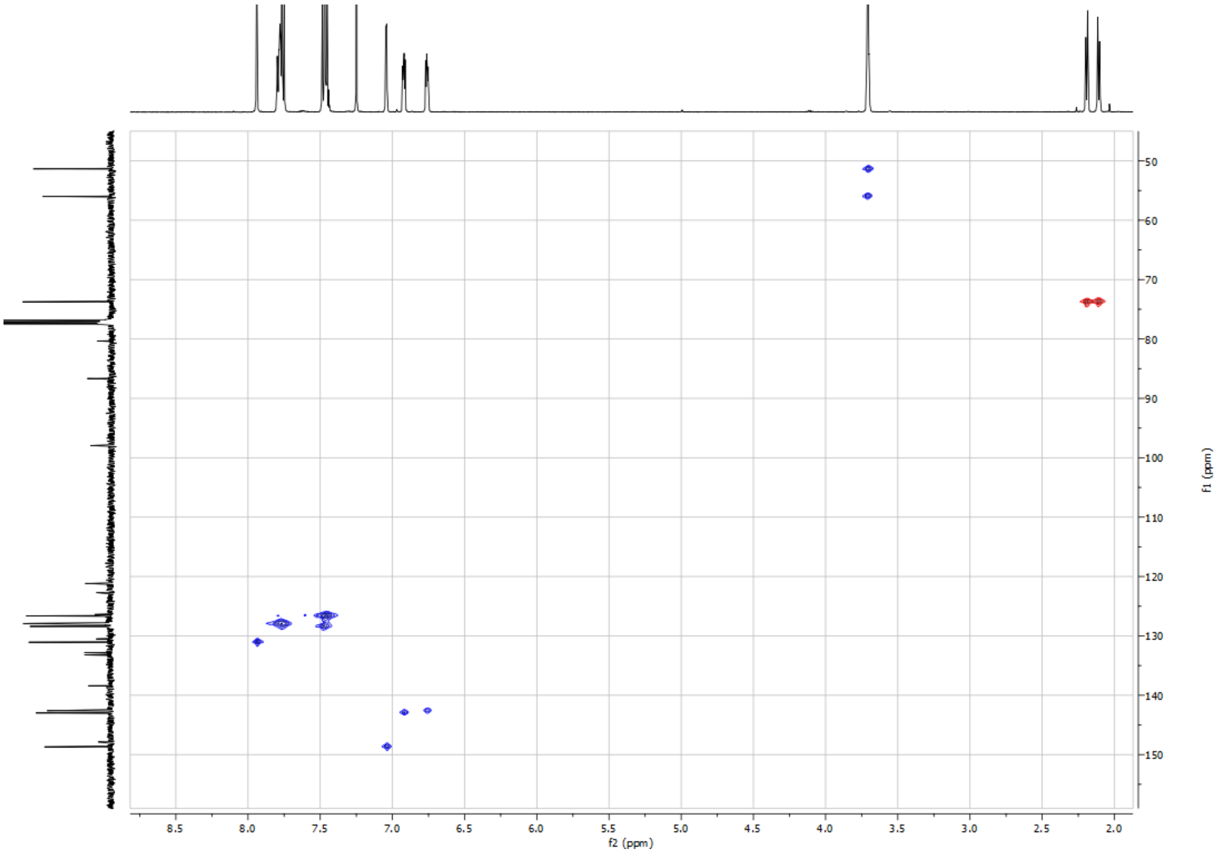

Figure S15: HSQC NMR spectrum (500 MHz) of **1i** in $CDCl_3$.

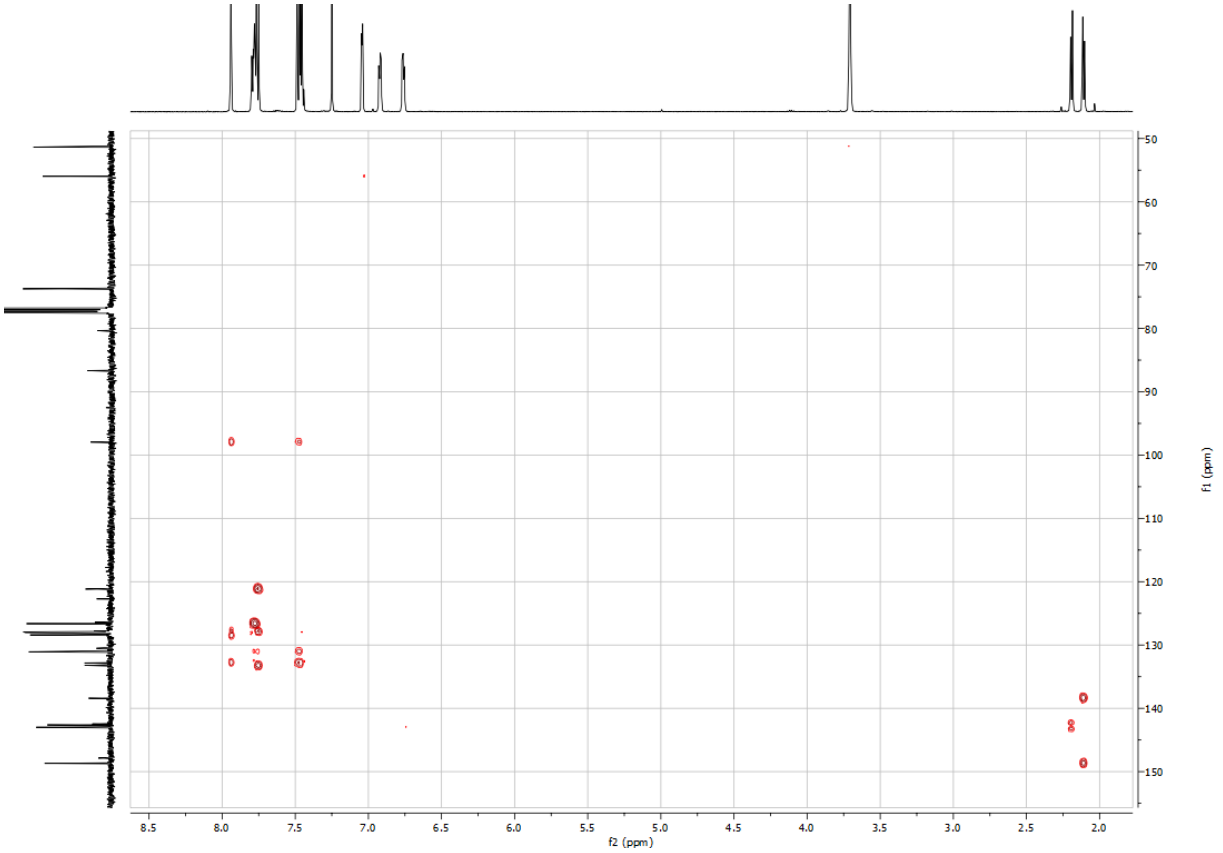

Figure S16: HMBC NMR spectrum (500 MHz) of **1i** in $CDCl_3$.

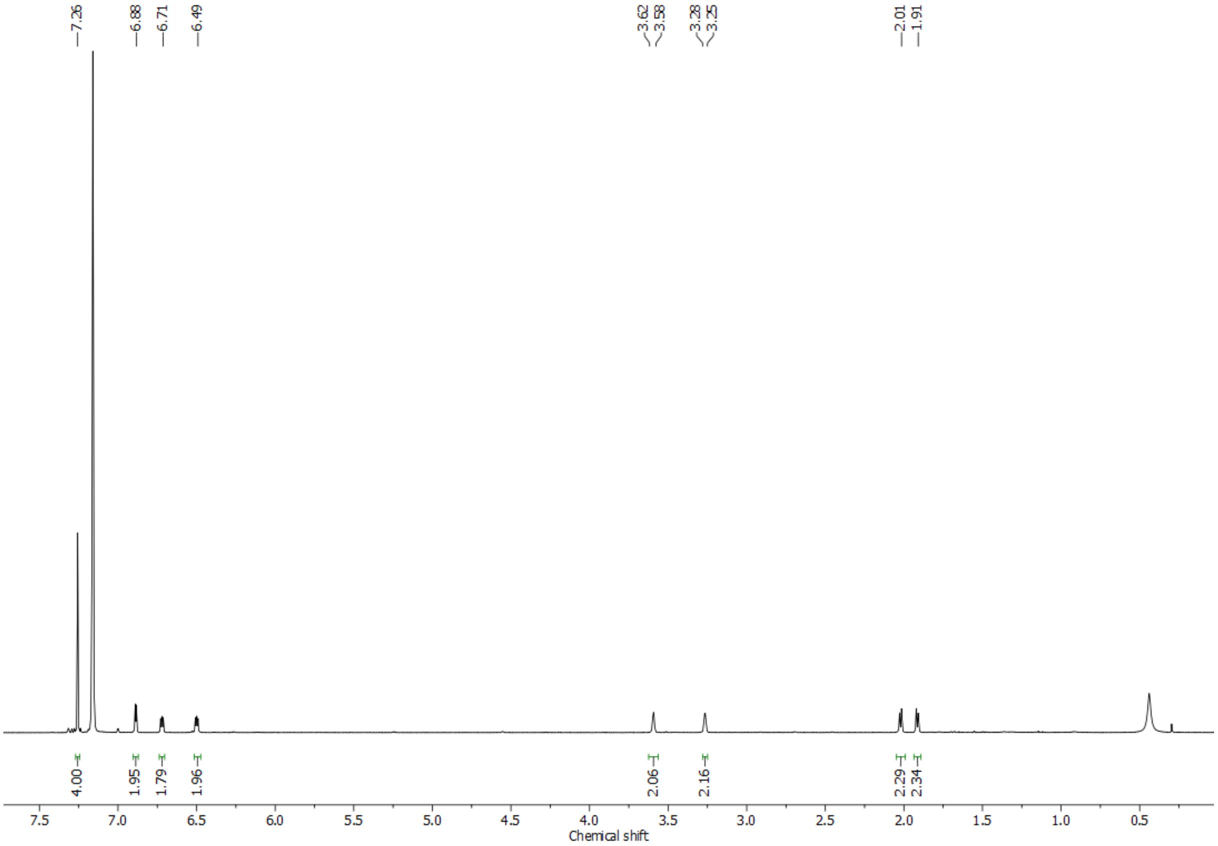

Figure S17: ^{1}H NMR spectrum (500 MHz) of **1j** in benzene-d_6.

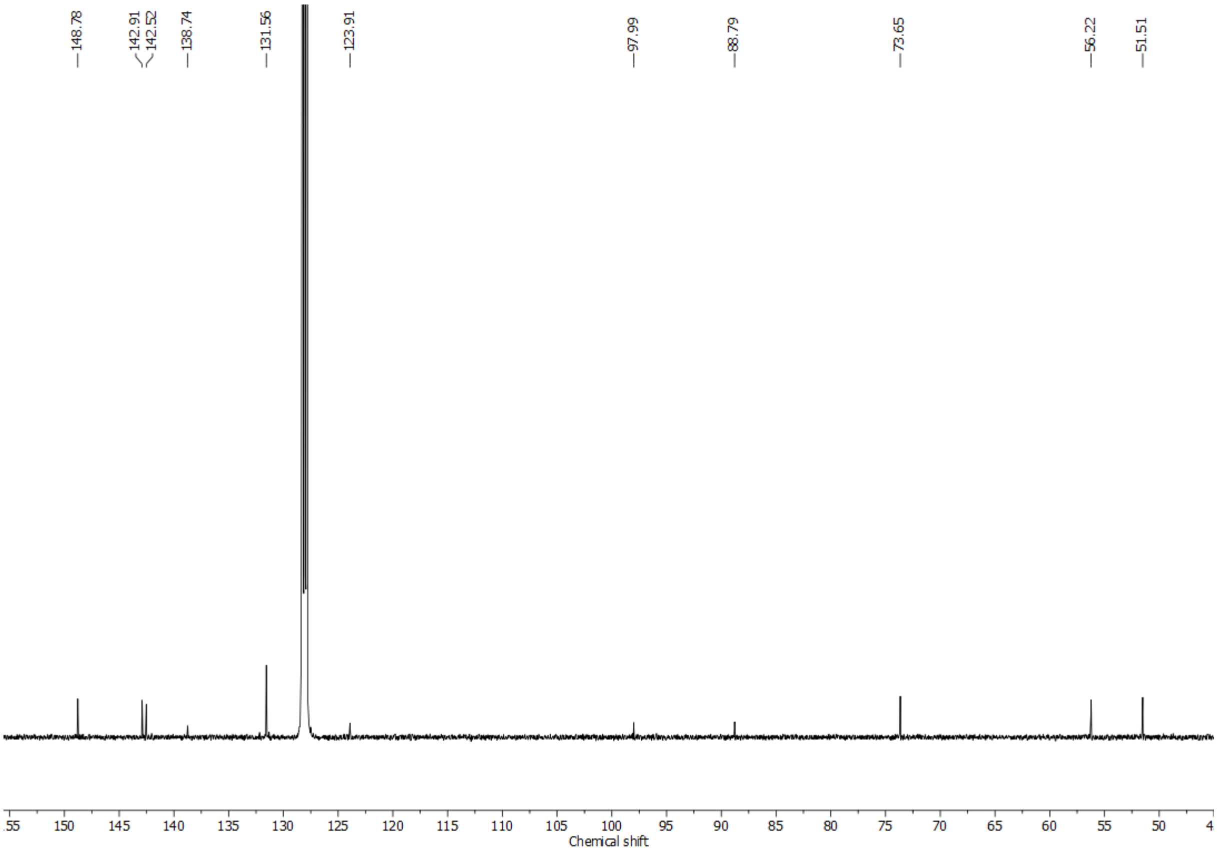

Figure S18: ^{13}C NMR spectrum (125 MHz) of **1j** in benzene-d_6.

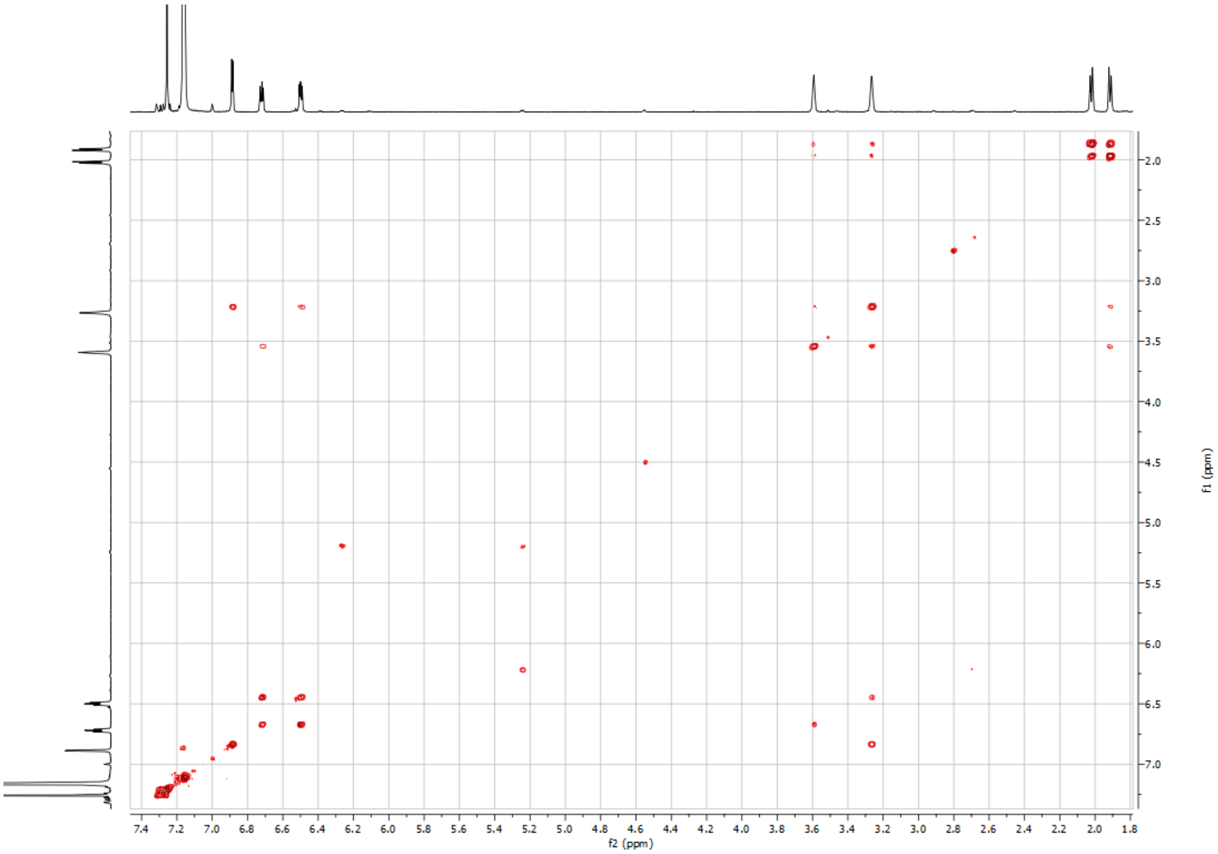

Figure S19: H,H-COSY NMR spectrum (500 MHz) of **1j** in benzene-d_6.

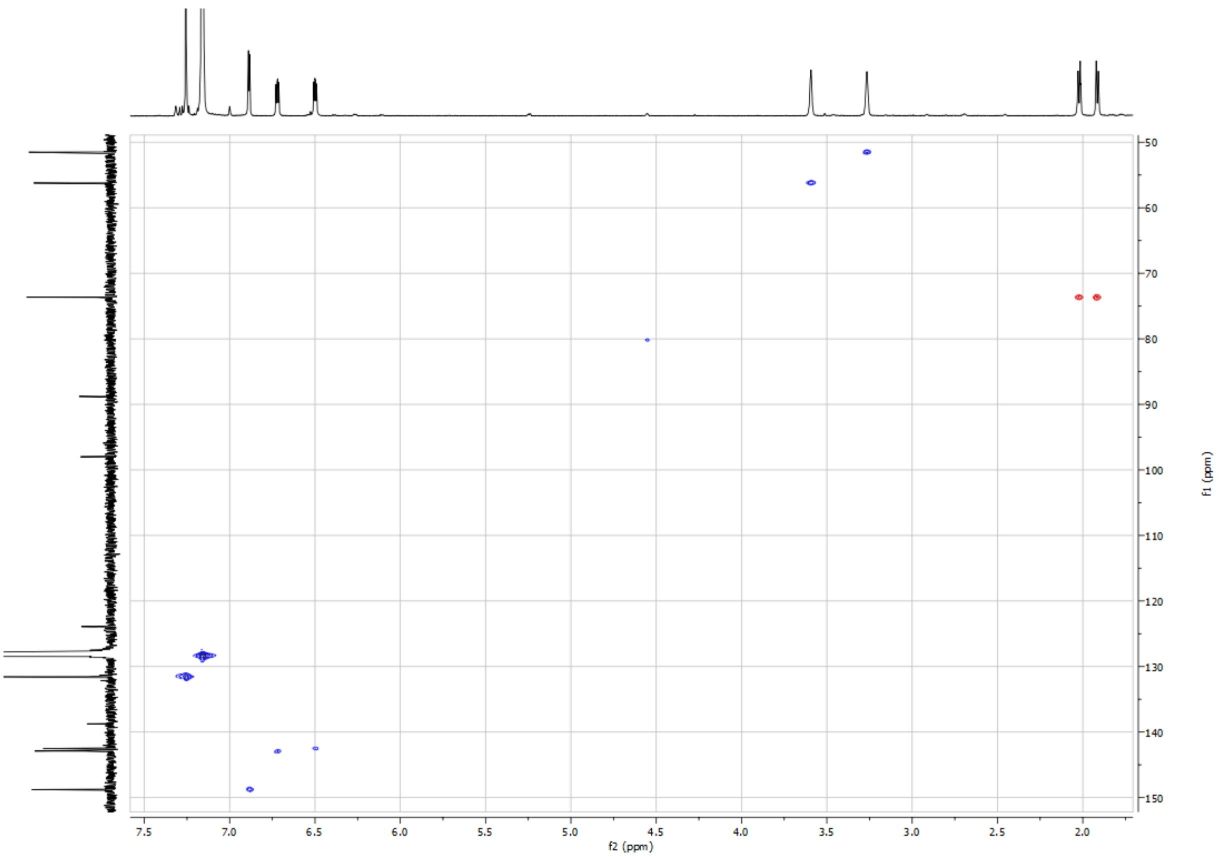

Figure S20: HSQC NMR spectrum (500 MHz) of **1j** in benzene-d_6.

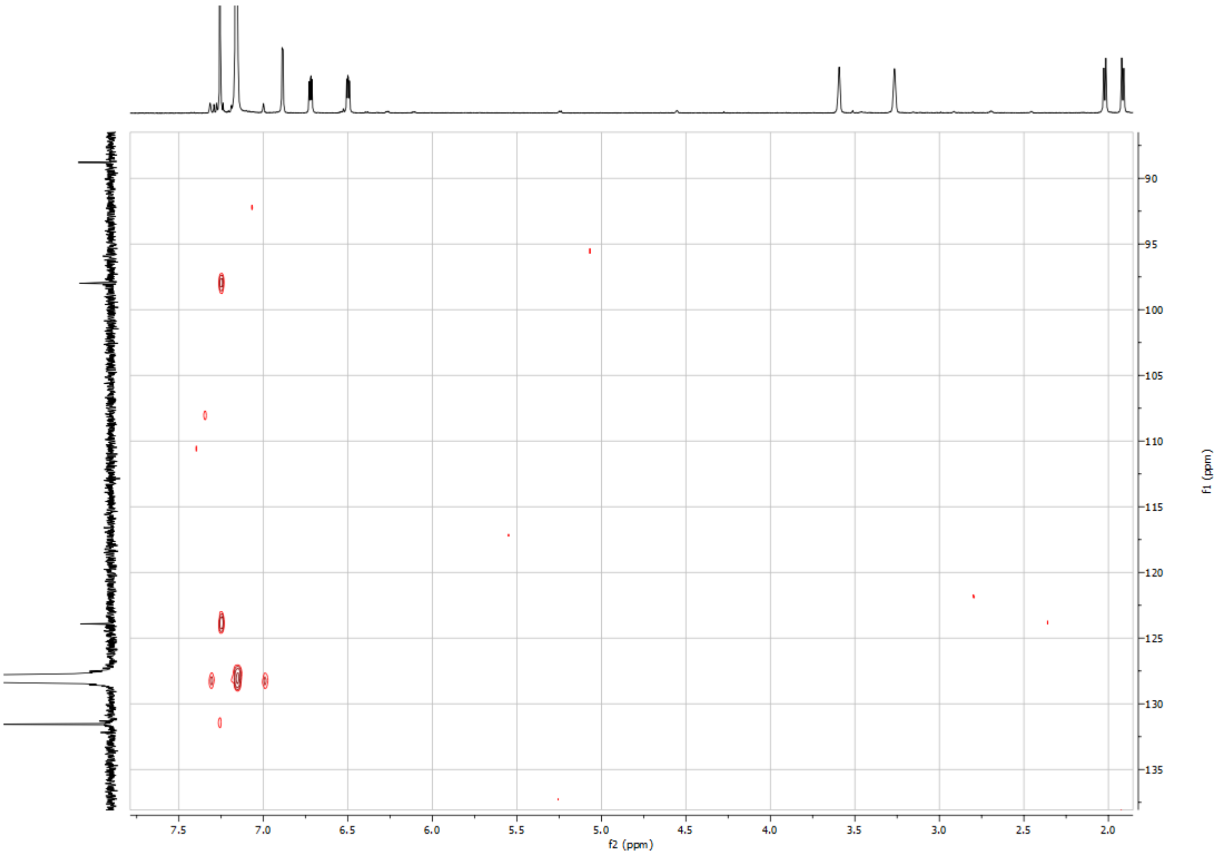

Figure S21: HMBC NMR spectrum (500 MHz) of **1j** in benzene-d_6.

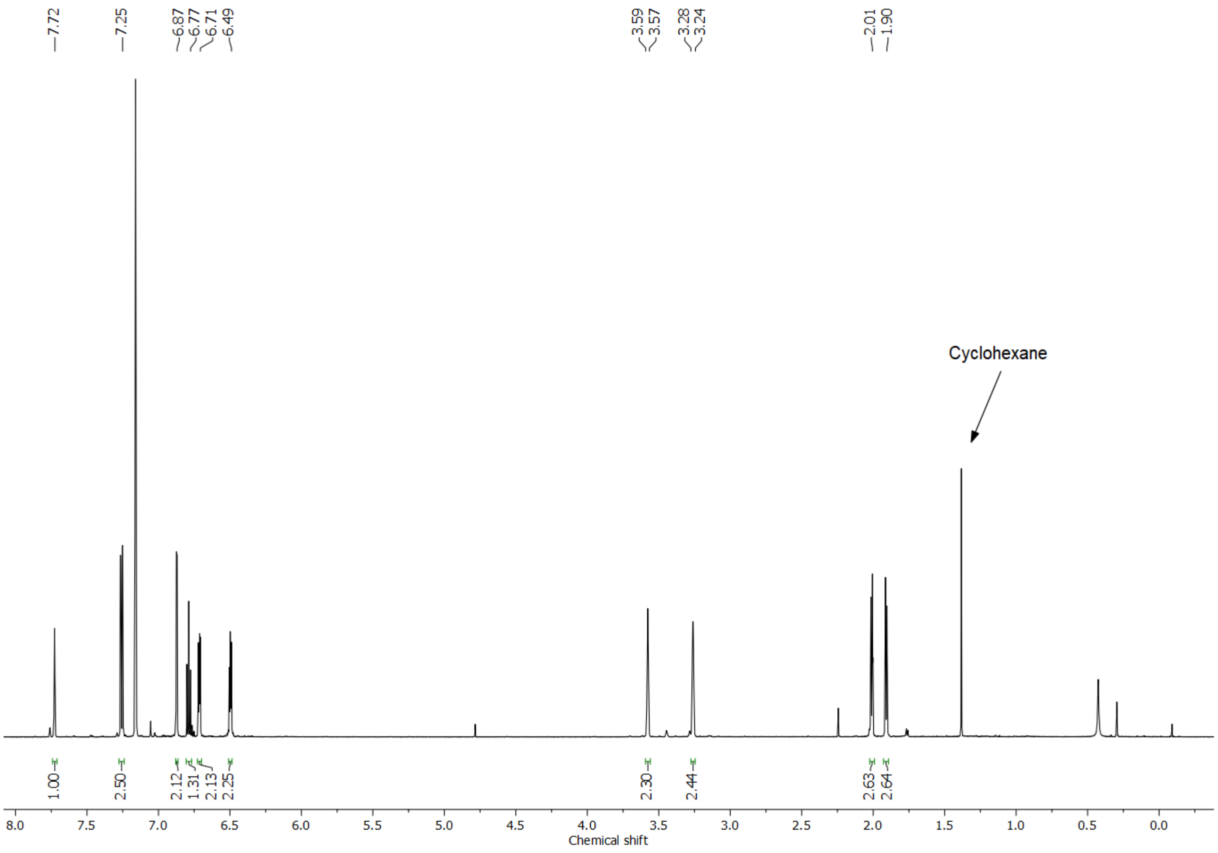

Figure S22: ^{1}H NMR spectrum (500 MHz) of **1k** in benzene-d_6.

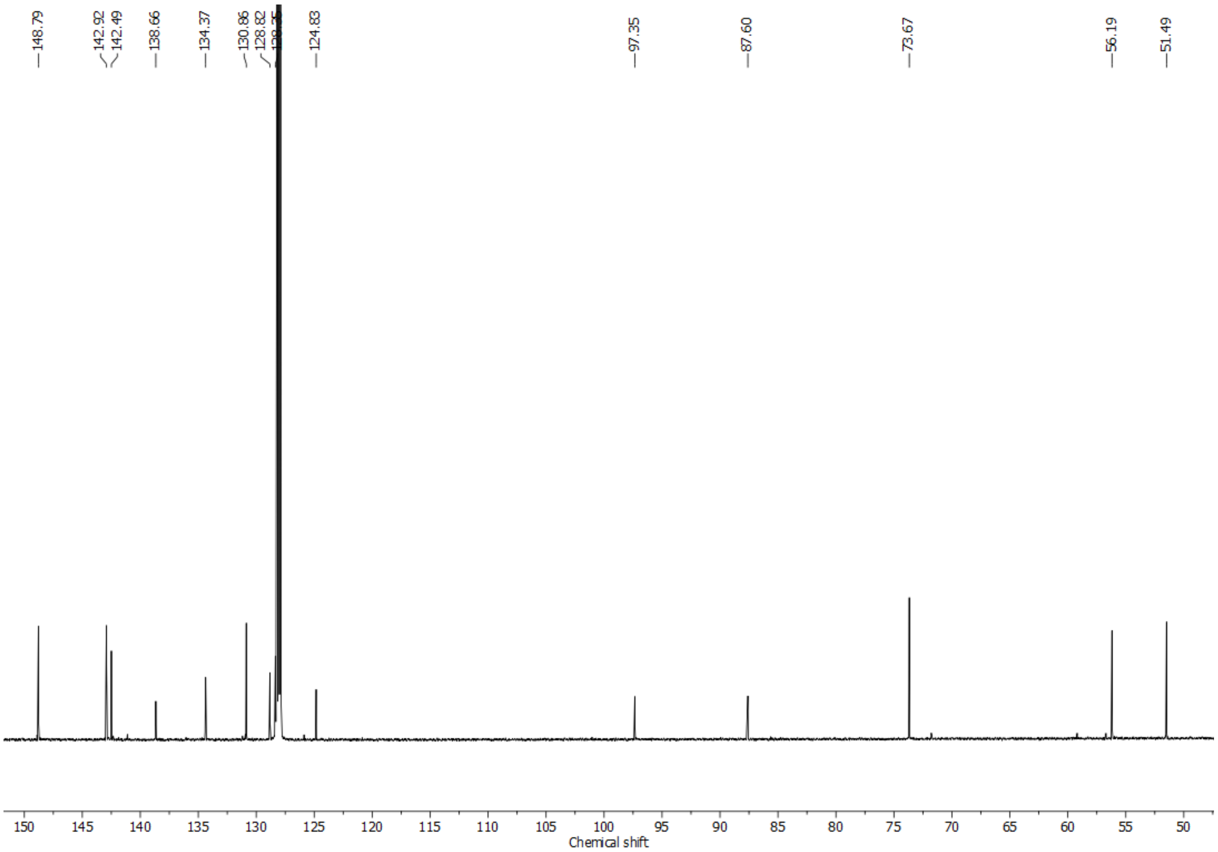

Figure S23: ^{13}C NMR spectrum (125 MHz) of **1k** in benzene-d_6.

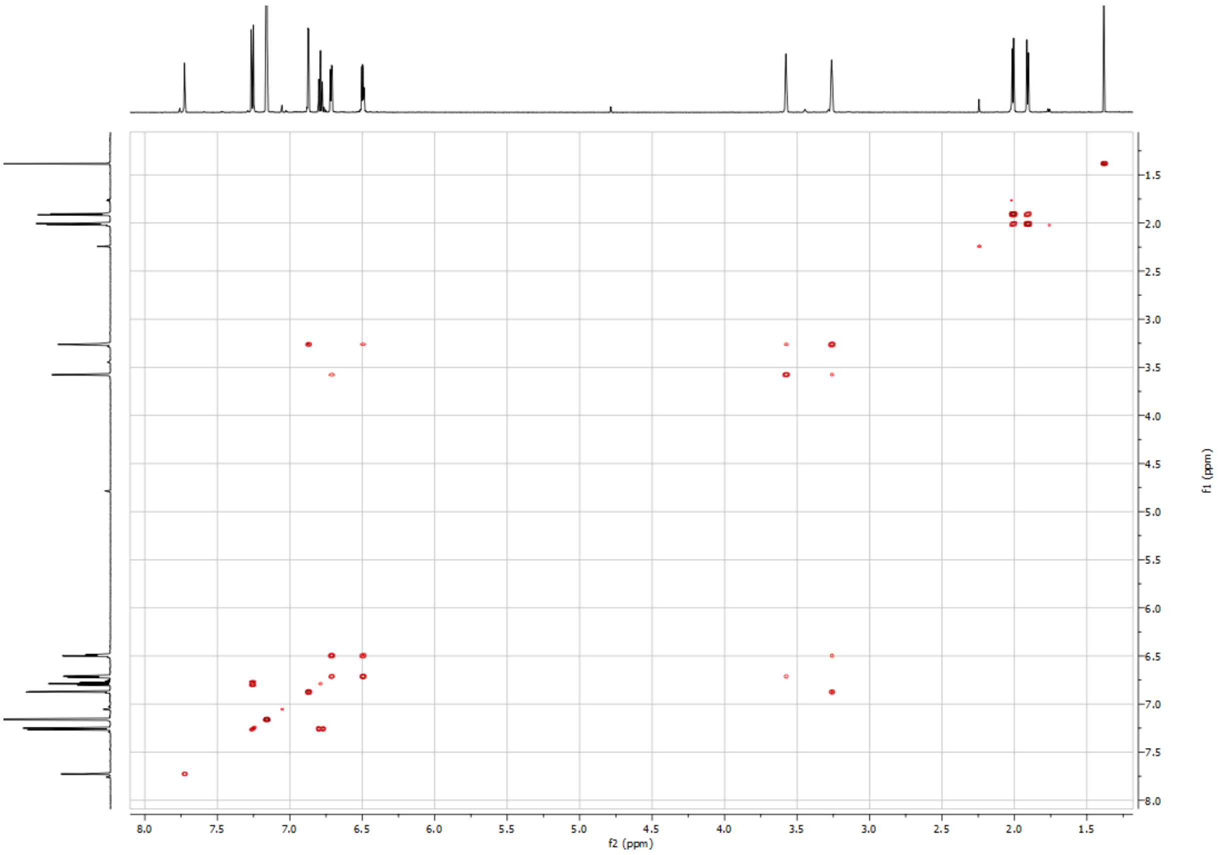

Figure S24: H,H-COSY NMR spectrum (500 MHz) of **1k** in benzene-d_6.

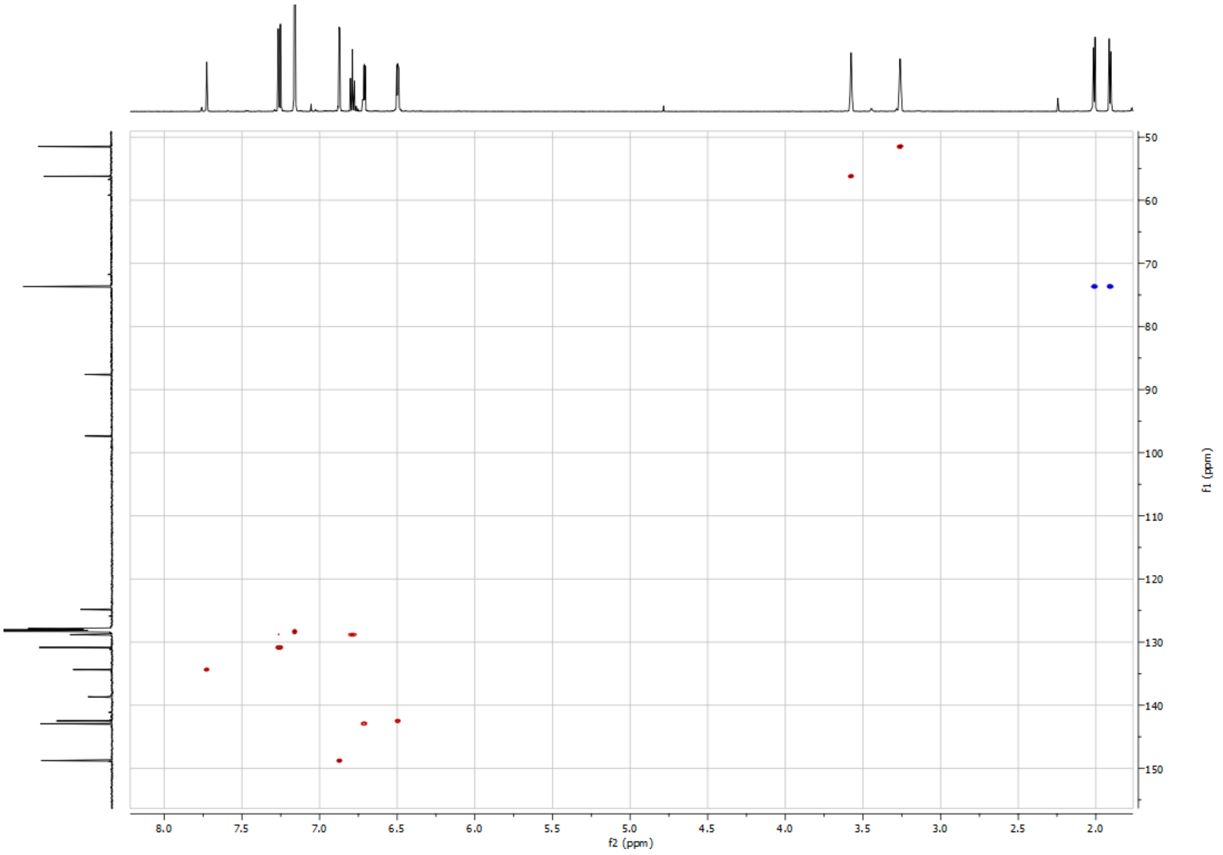

Figure S25: HSQC NMR spectrum (500 MHz) of **1k** in benzene-d_6.

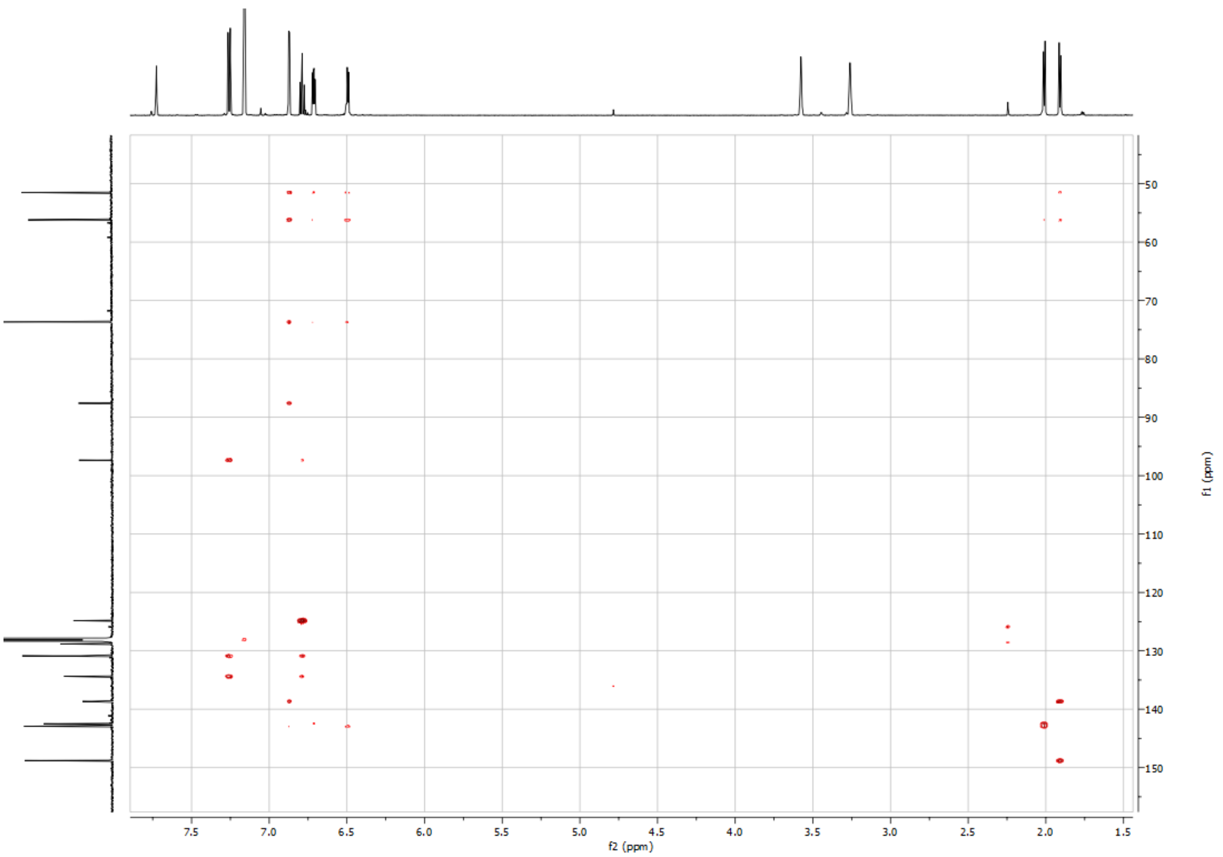

Figure S26: HMBC NMR spectrum (500 MHz) of **1k** in benzene-d_6.

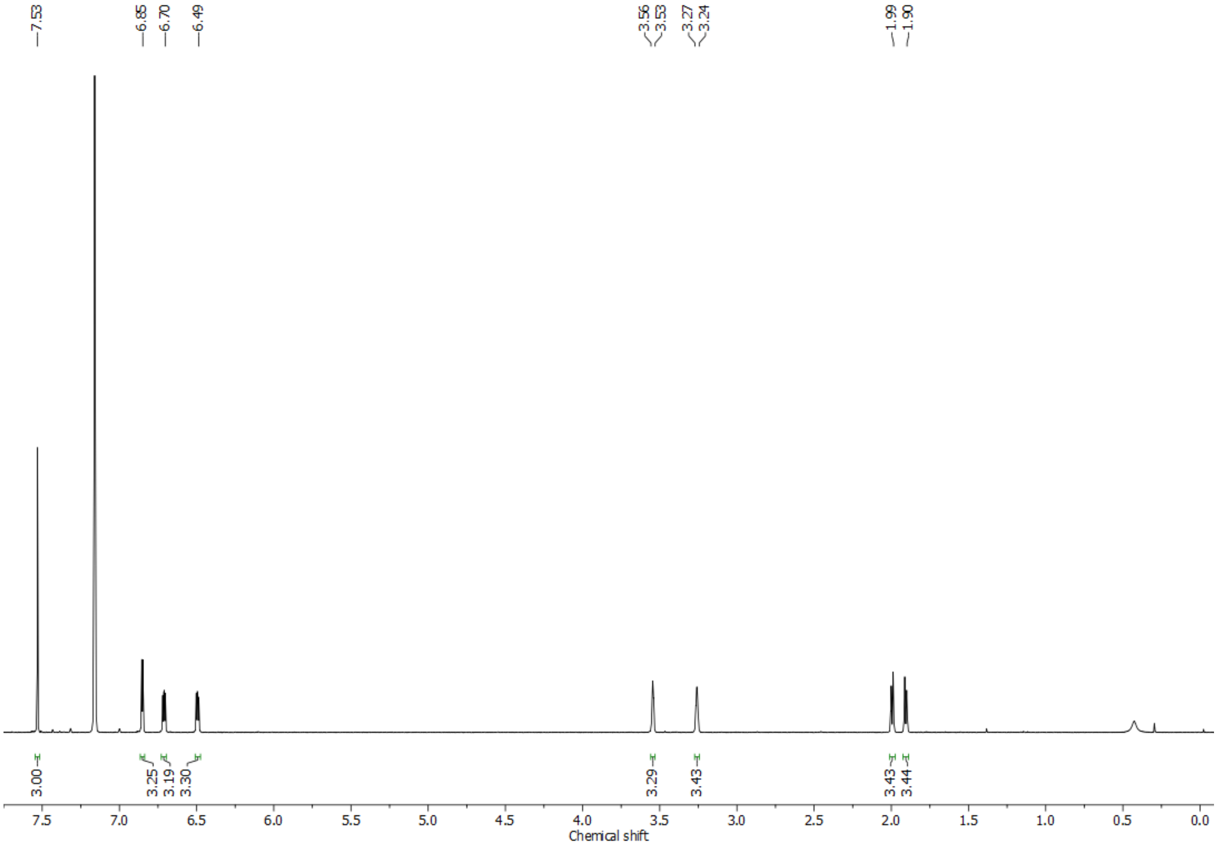

Figure S27: ^{1}H NMR spectrum (500 MHz) of **1l** in benzene-d_6.

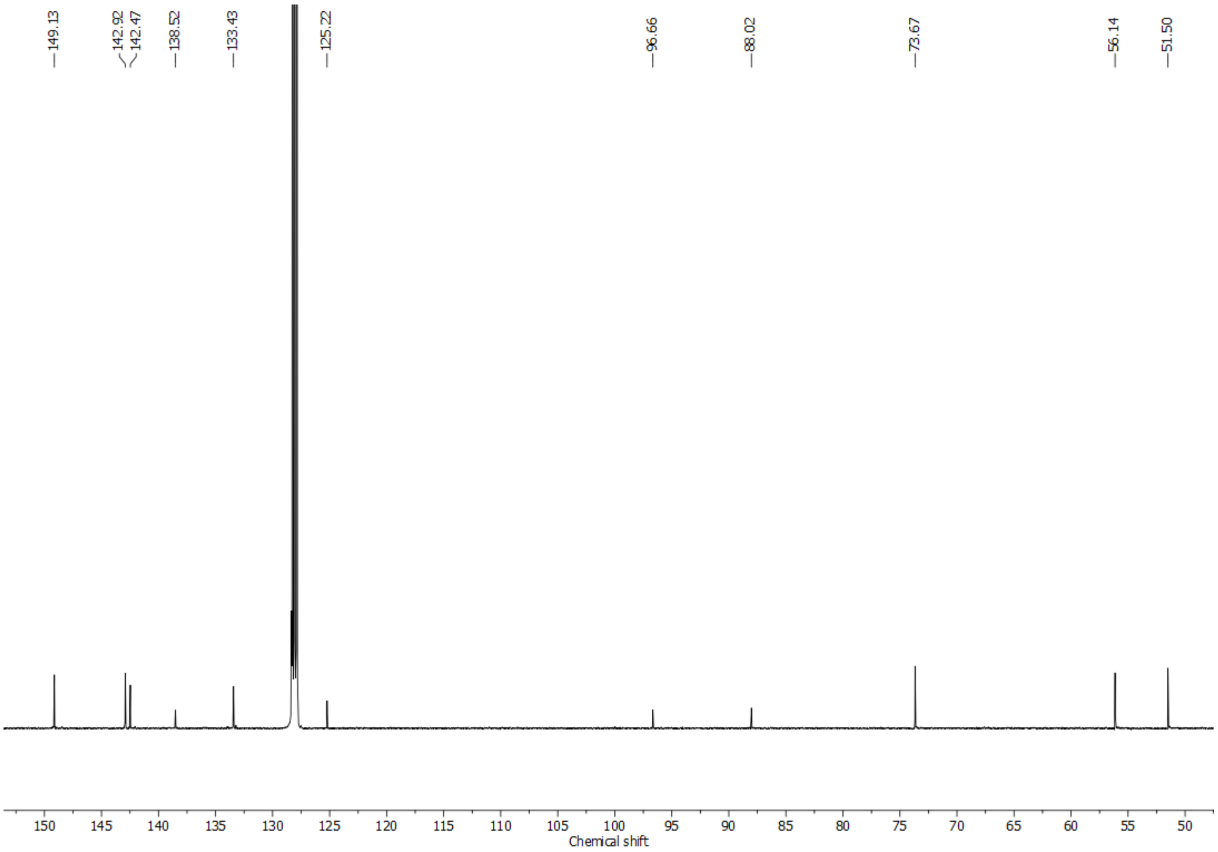

Figure S28: ^{13}C NMR spectrum (125 MHz) of **1l** in benzene-*d*6.

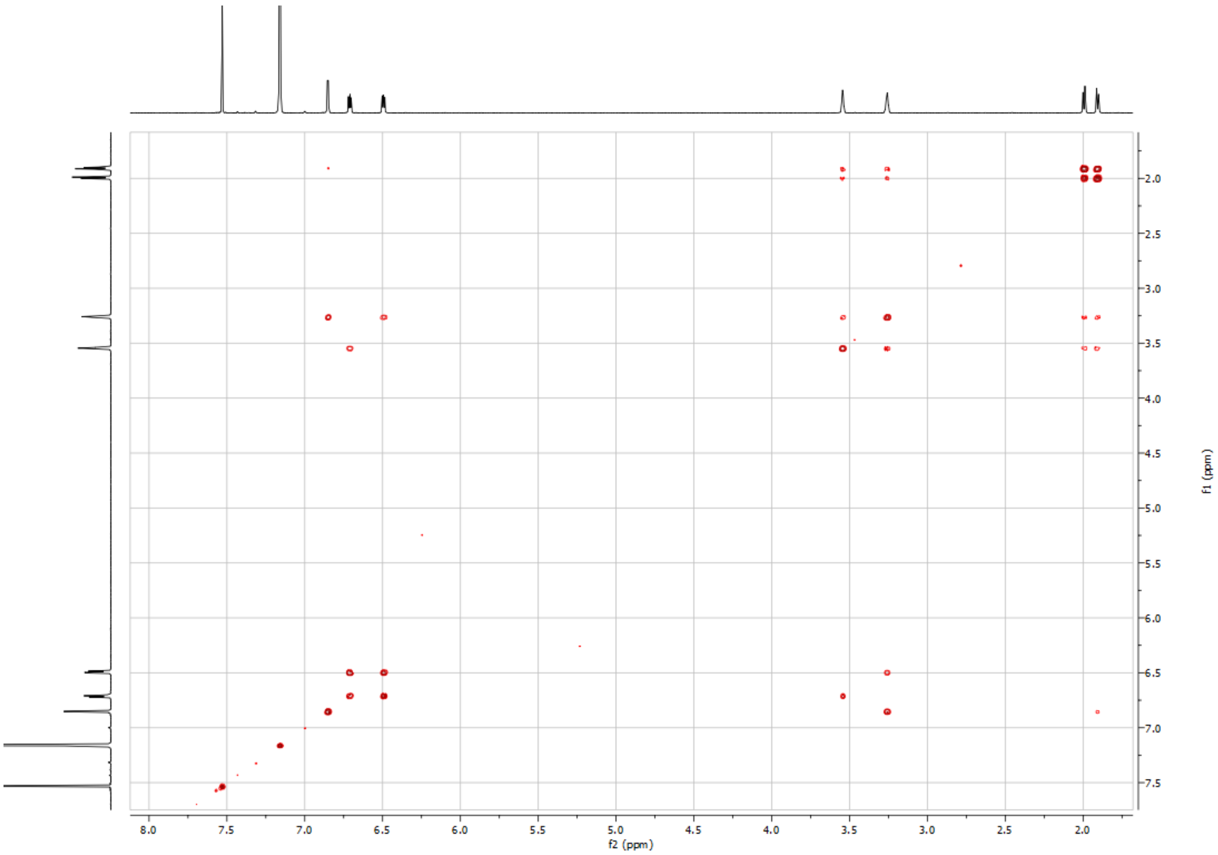

Figure S29: H,H-COSY NMR spectrum (500 MHz) of **1l** in benzene-*d*6.

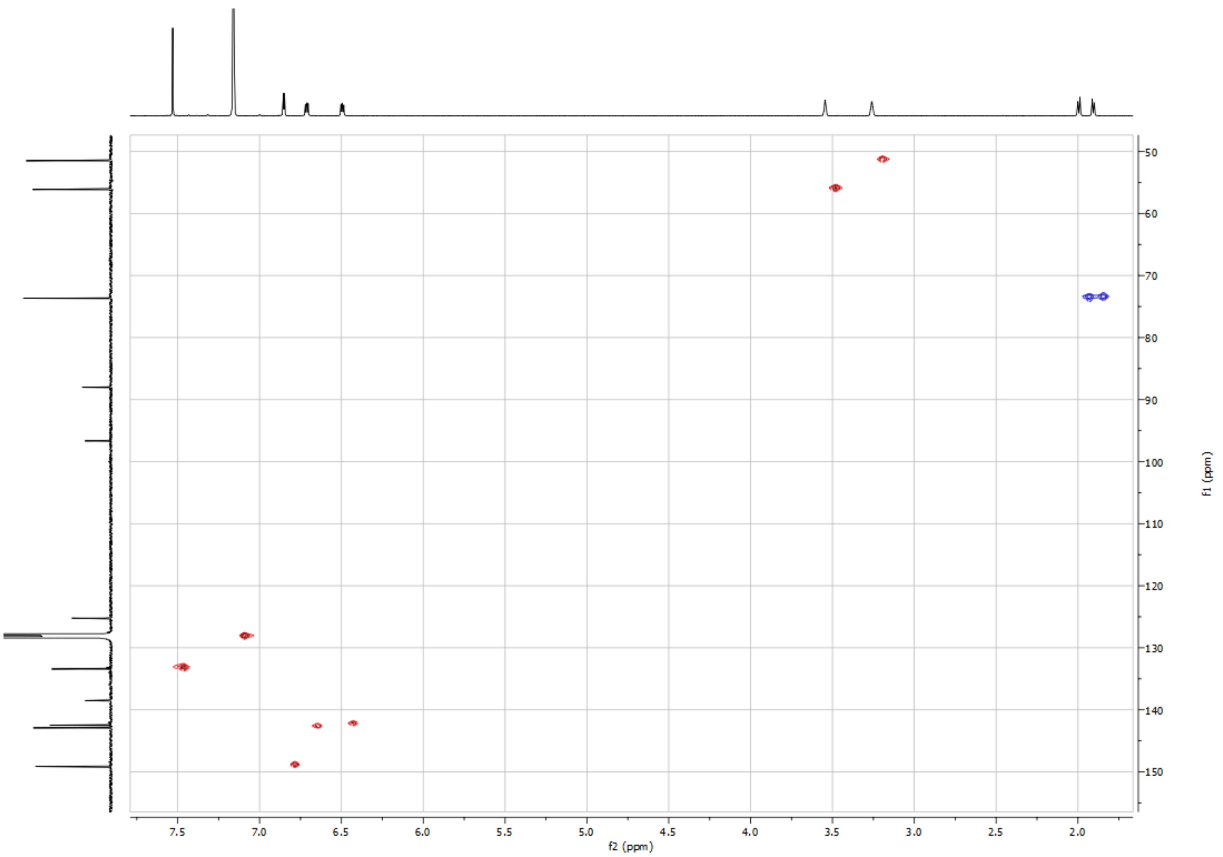

Figure S30: HSQC NMR spectrum (500 MHz) of **1l** in benzene-*d*6.

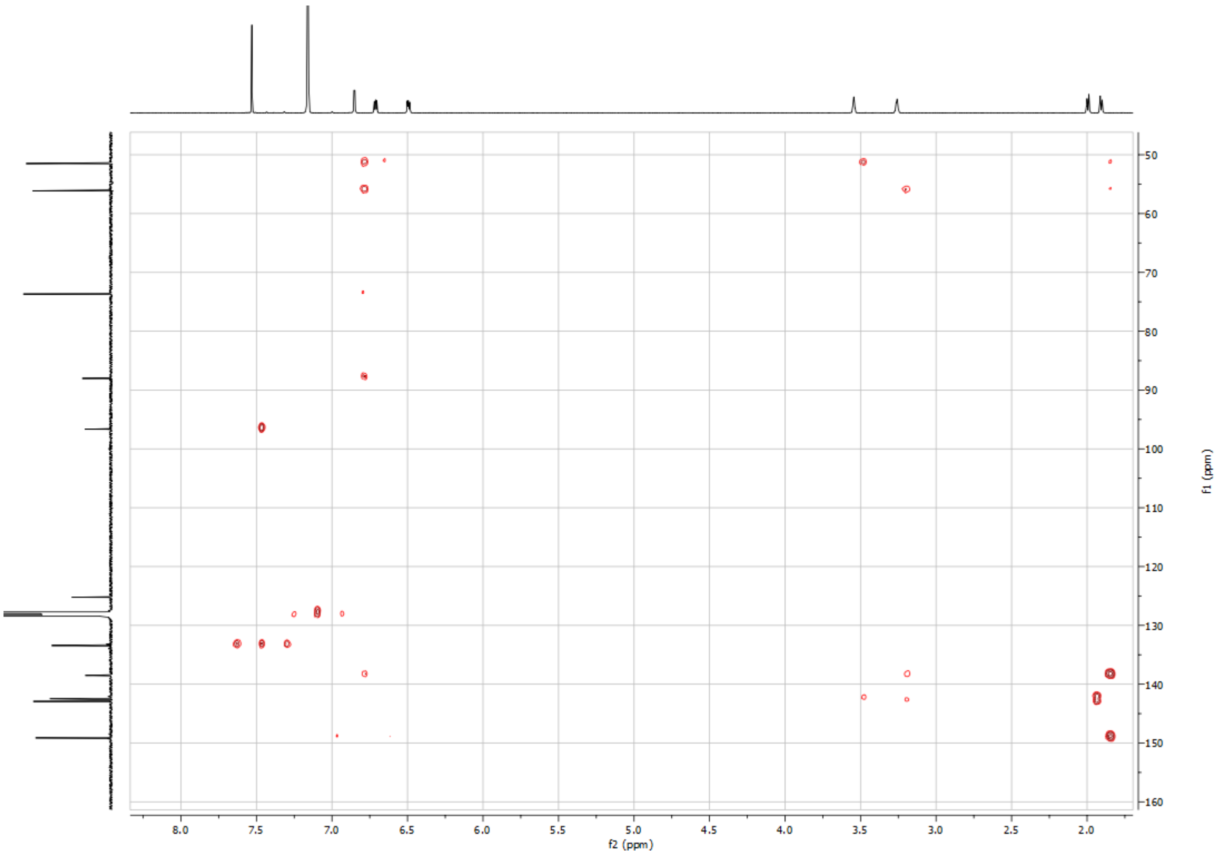

Figure S31: HMBC NMR spectrum (500 MHz) of **1l** in benzene-*d*6.

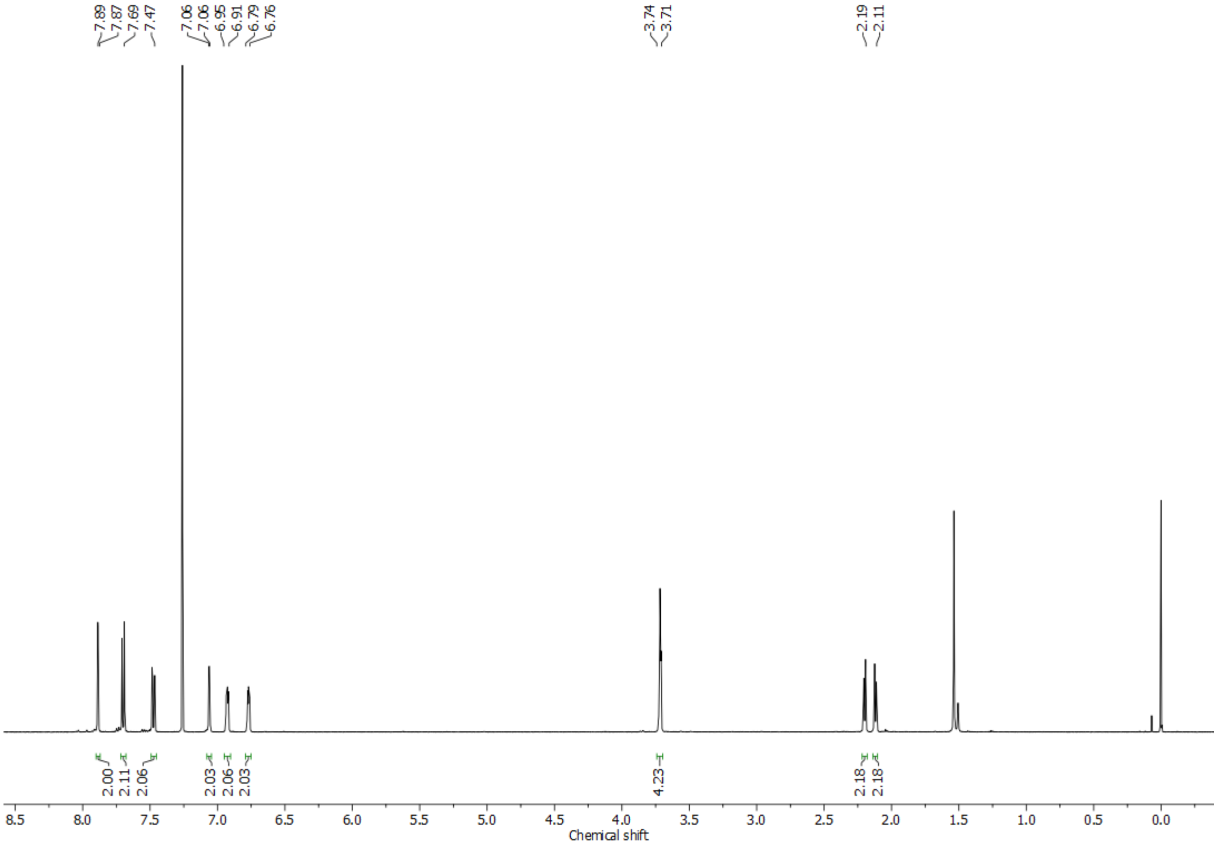

Figure S32: ^{1}H NMR spectrum (500 MHz) of **1n** in $CDCl_3$.

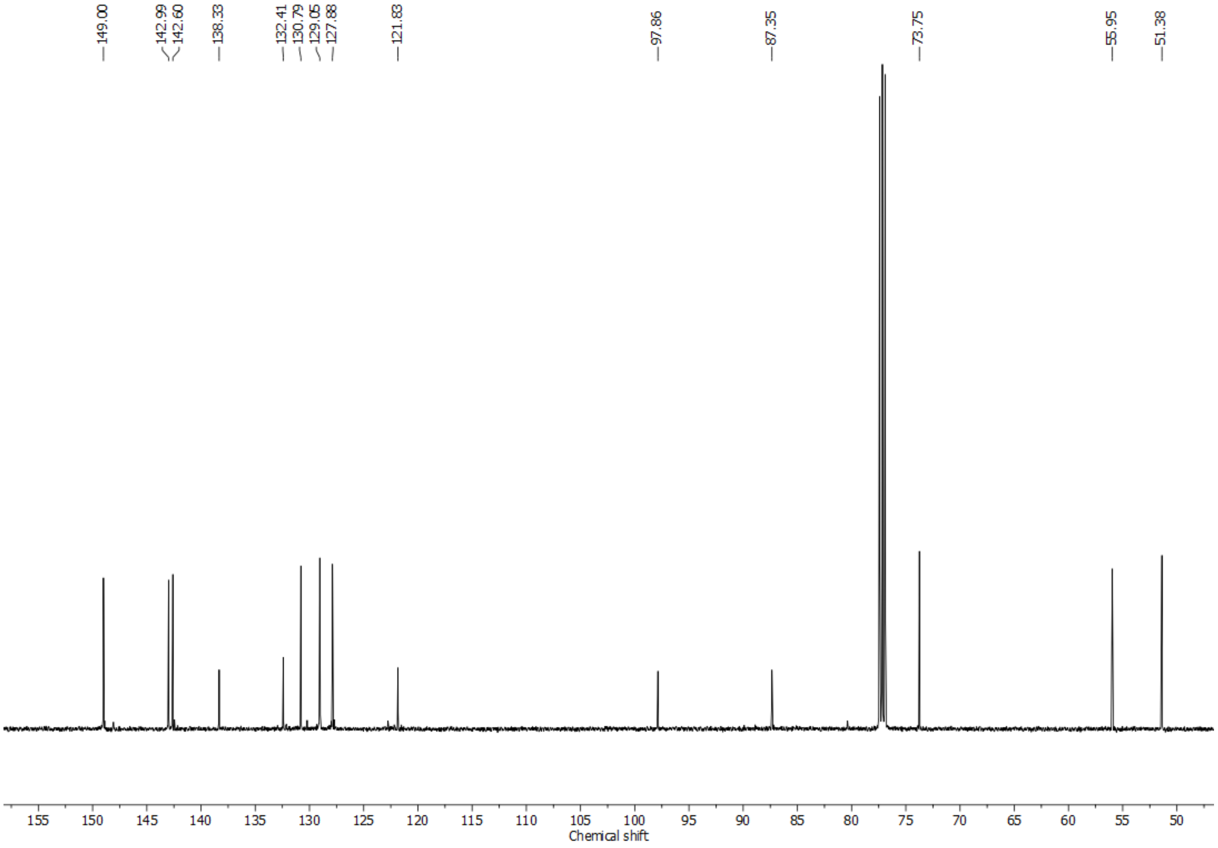

Figure S33: ^{13}C NMR spectrum (125 MHz) of **1n** in $CDCl_3$.

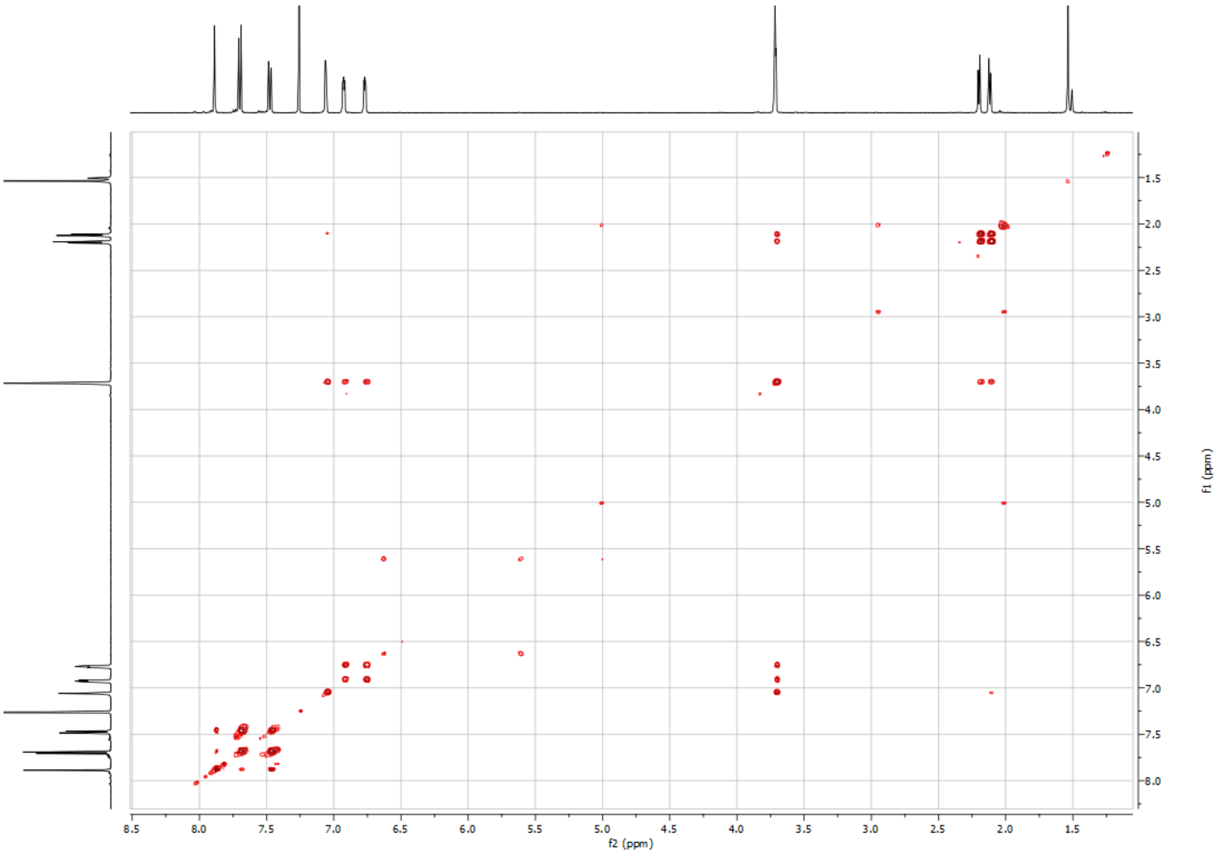

Figure S34: H,H-COSY NMR spectrum (500 MHz) of **1n** in $CDCl_3$.

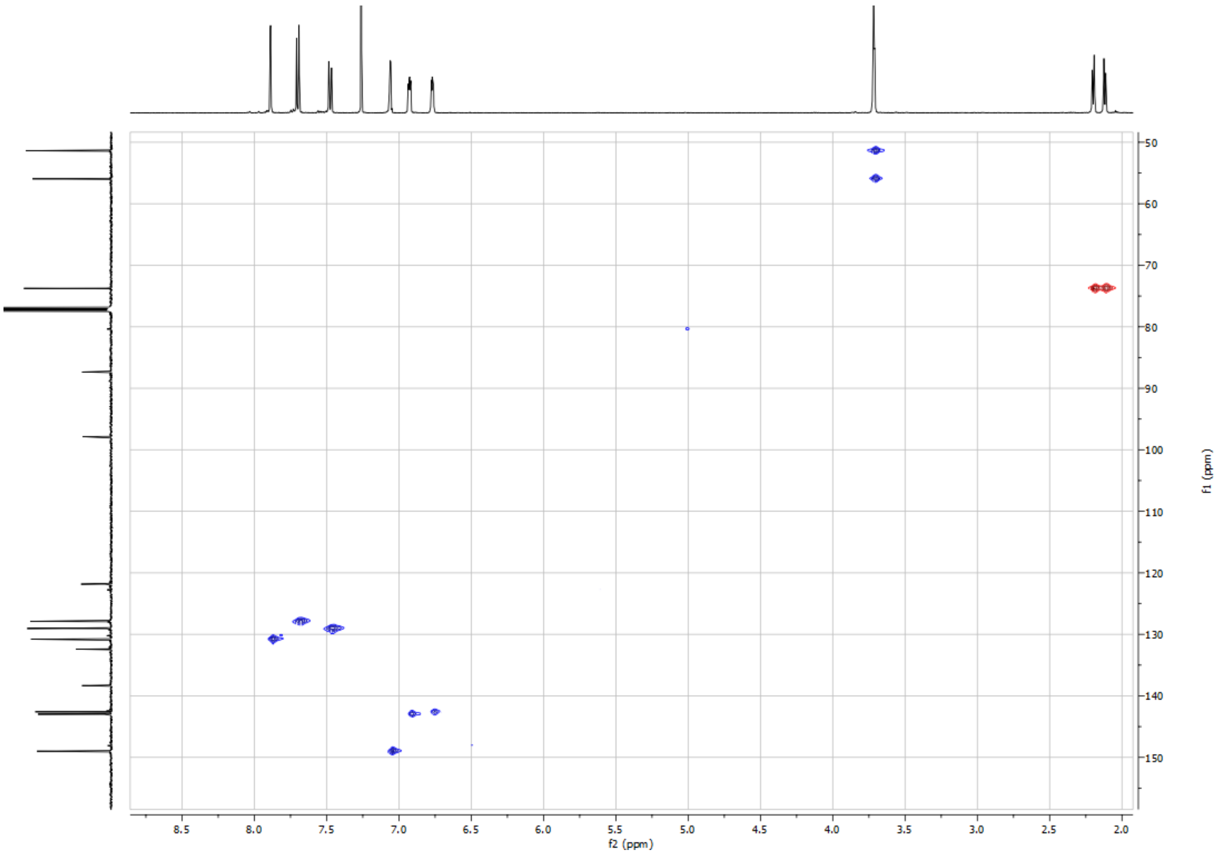

Figure S35: HSQC NMR spectrum (500 MHz) of **1n** in $CDCl_3$.

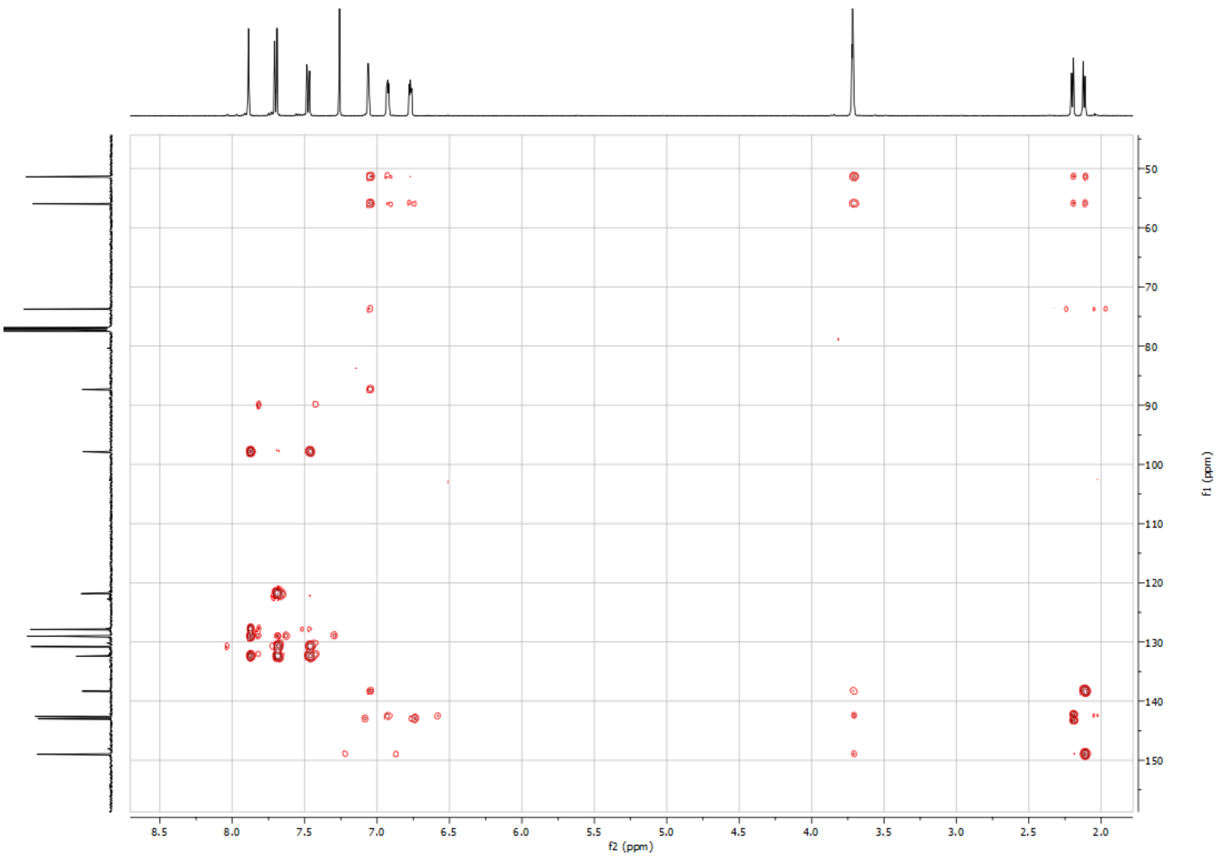

Figure S36: HMBC NMR spectrum (500 MHz) of **1n** in $CDCl_3$.

S9.2 NMR-spectroscopic monitoring of the photoreaction

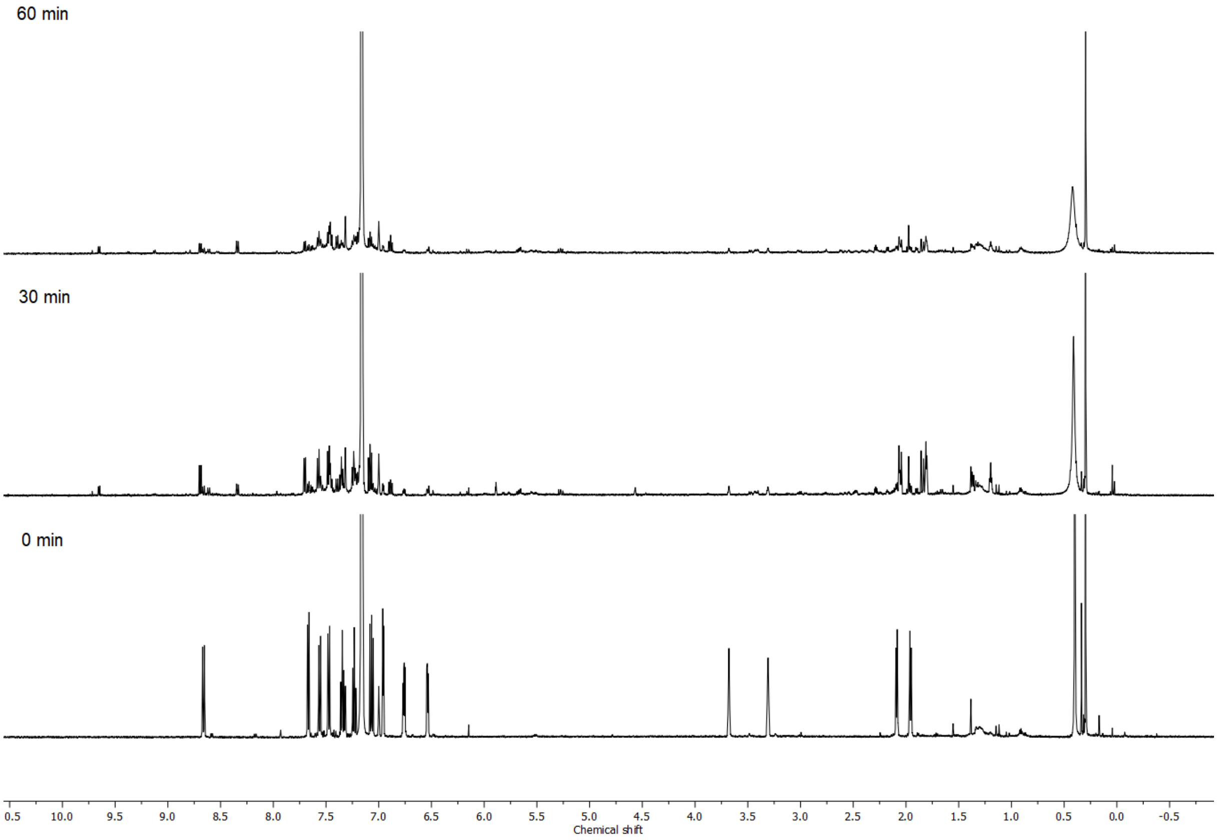

Figure S37: ^{1}H NMR spectra (500 MHz) of **1h** in benzene-d_6 before and after irradiation with λ_{ex} = 340 nm. Irradiation outside the NMR spectrometer.

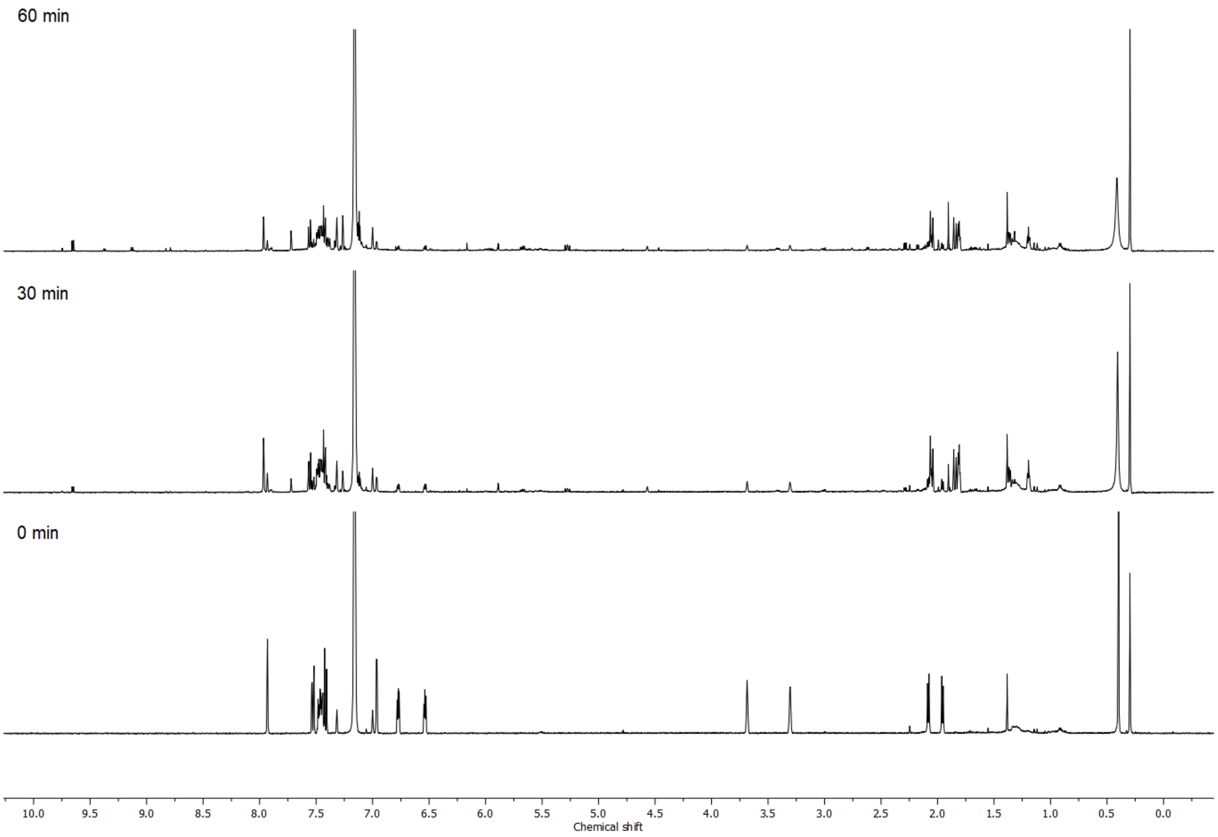

Figure S38: ^{1}H NMR spectra (500 MHz) of **1i** in benzene-*d*6 before and after irradiation with λ_{ex} = 310 nm. Irradiation outside the NMR spectrometer.

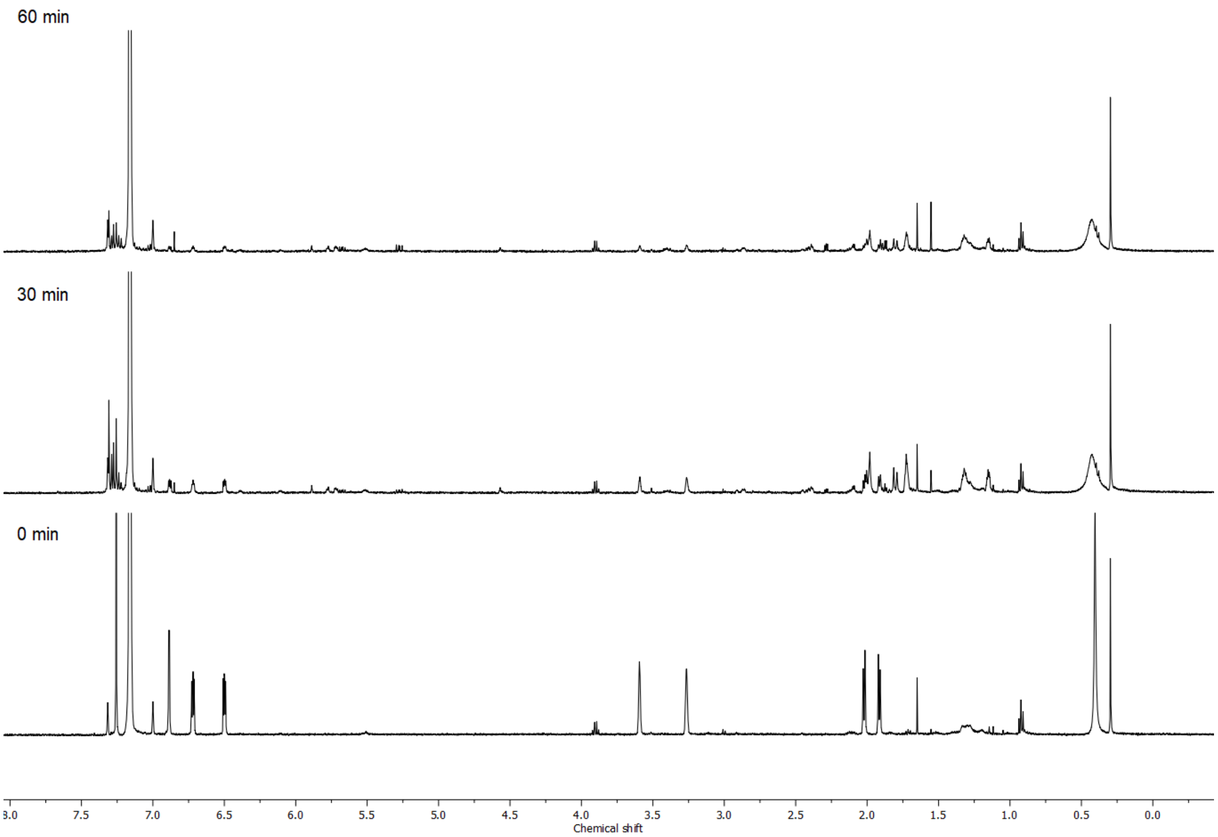

Figure S39: ^{1}H NMR spectra (500 MHz) of **1j** in benzene-*d*6 before and after irradiation with λ_{ex} = 340 nm. Irradiation outside the NMR spectrometer.

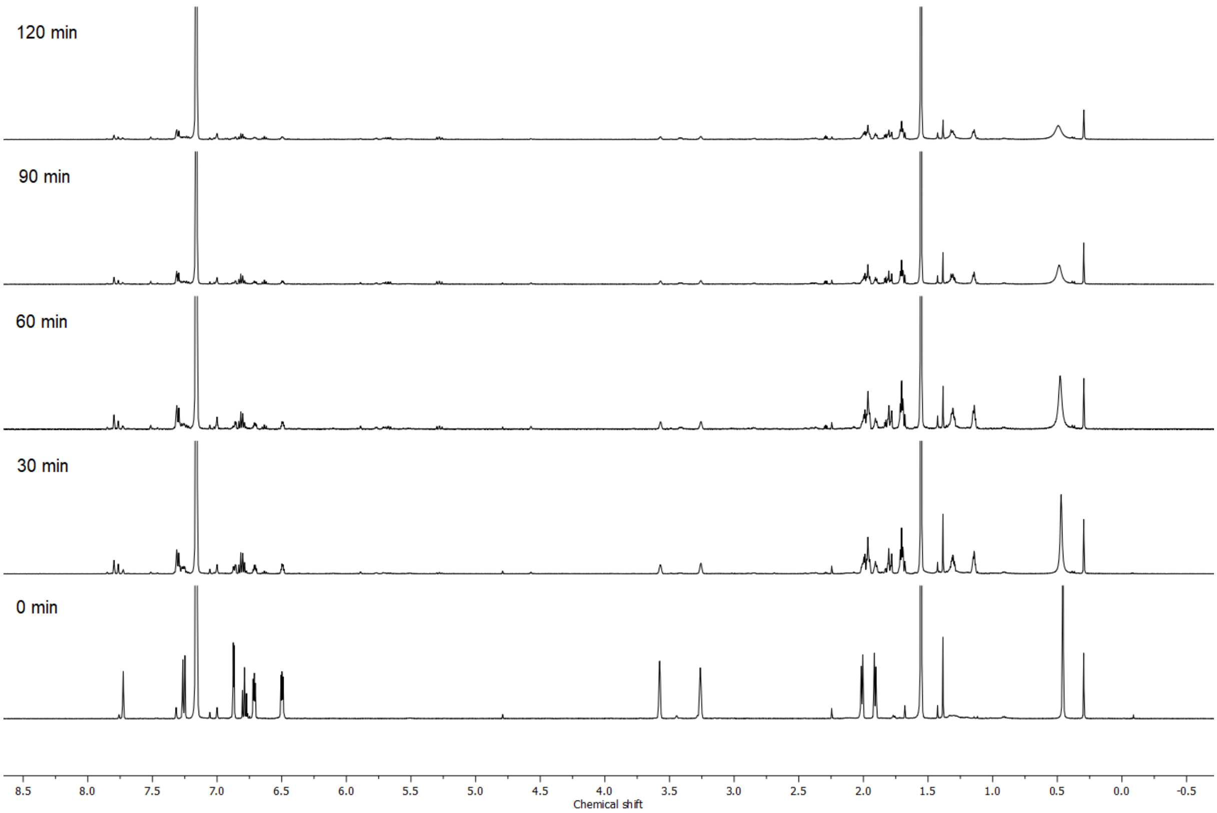

Figure S40: ^{1}H NMR spectra (500 MHz) of **1k** in benzene-*d*$_6$ before and after irradiation with λ_{ex} = 310 nm. Irradiation outside the NMR spectrometer.

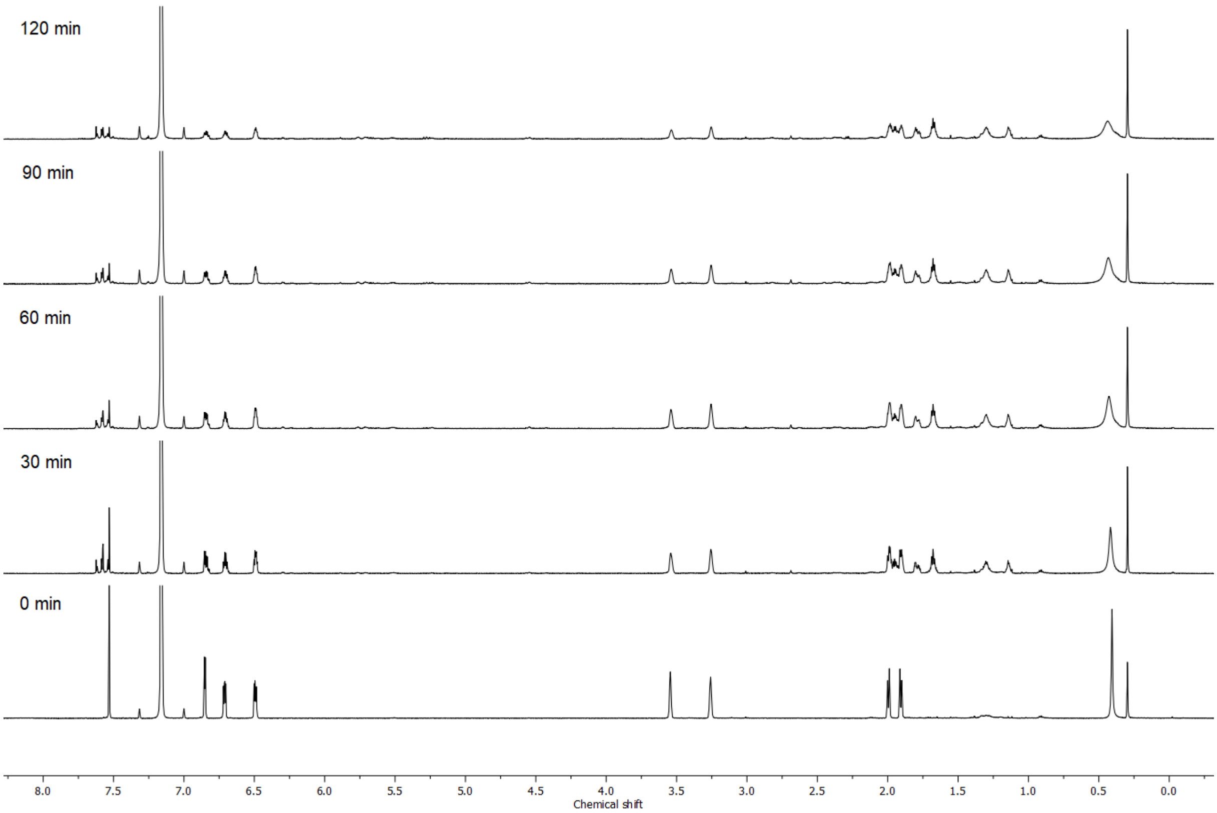

Figure S41: ^{1}H NMR spectra (500 MHz) of **1l** in benzene-*d*$_6$ before and after irradiation with λ_{ex} = 310 nm. Irradiation outside the NMR spectrometer.

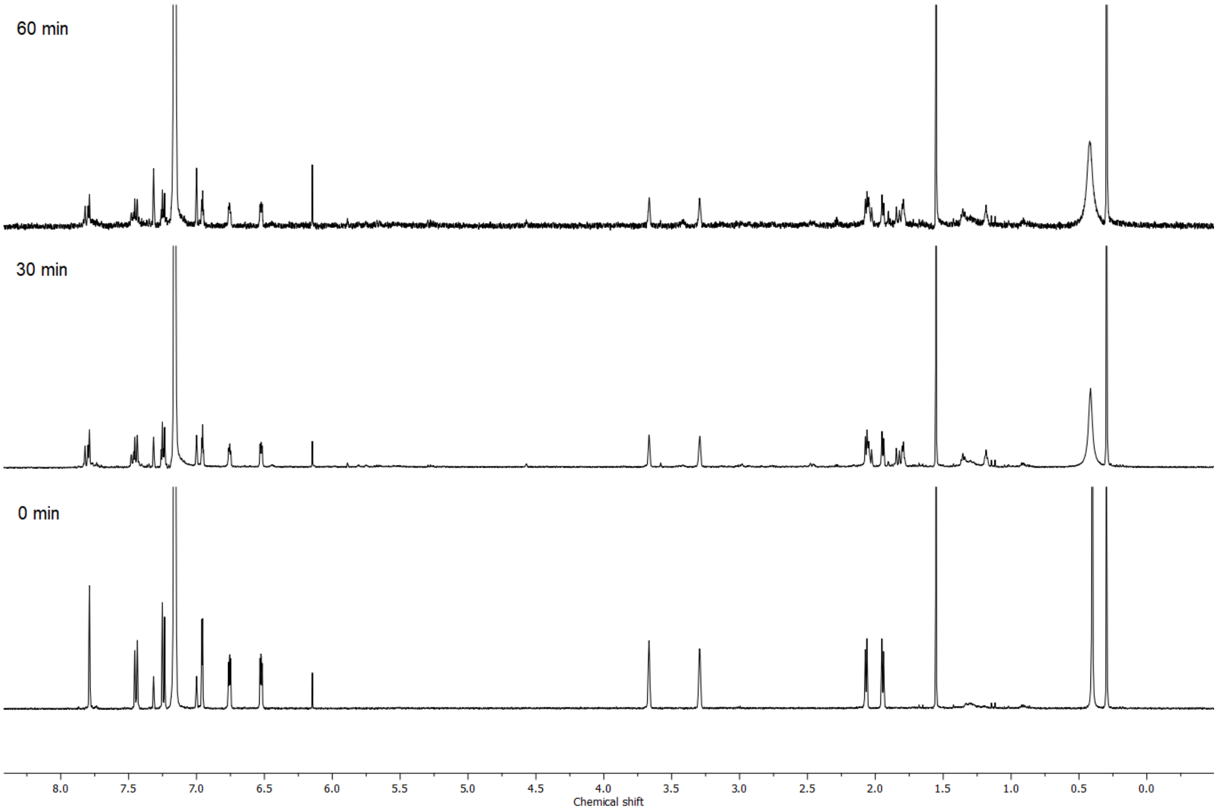

Figure S42: ^{1}H NMR spectra (500 MHz) of **1n** in benzene-d_6 before and after irradiation with λ_{ex} = 340 nm. Irradiation outside the NMR spectrometer.

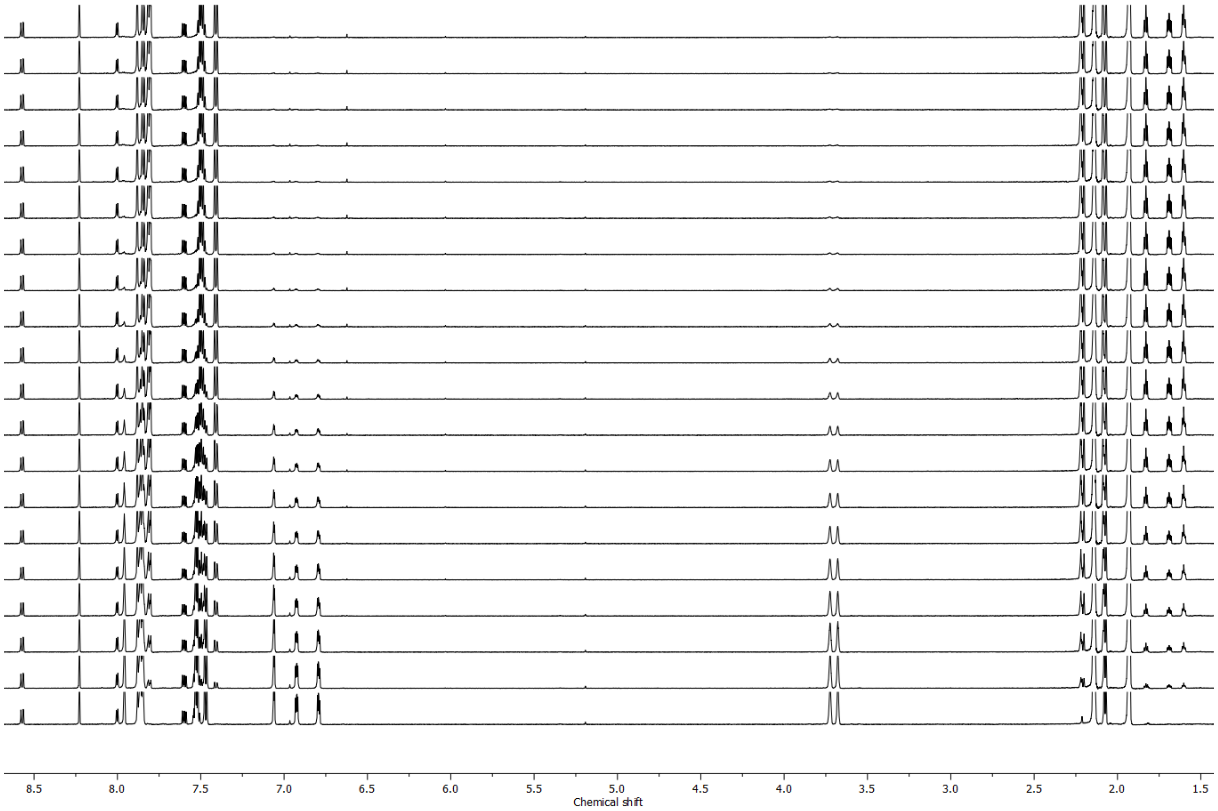

Figure S43: ^{1}H NMR spectra (600 MHz) of **1i** in the presence of [Ru(phen)$_3$(PF$_6$)$_2$] mecn-d_3 after varying irradiation times with λ_{ex} = 520 nm. In-situ NMR irradiation, spectra were recorded in intervals of 1 min and displayed for 20 min.

S10 References

[1] T. Paululat, M. Rabe, D. V. Berdnikova, *J. Magn. Reson.* **2021**, *327*, 106990.

[2] OriginLab Corporation, OriginPro 8.5.1, Northhampton, MA, USA, 2011.

[3] P. Peluso, C. Greco, O. D. Lucchi, S. Cossu, *Eur. J. Org. Chem.* **2002**, 4024.

[4] a) I. R. Butler, C. Soucy-Breau, *Can. J. Chem.* **1991**, *69*, 1117; b) K. Ohta, T. Goto, Y. Endo, *Inorg. Chem.* **2005**, *44*, 8569.

[5] a) B. Blanco, A. Sedes, A. Peón, H. Lamb, A. R. Hawkins, L. Castedo, C. González-Bello, *Org. Biomol. Chem.* **2012**, *10*, 3662; b) H. Uji, J. Ogawa, K. Itabashi, T. Imai, S. Kimura, *Chem. Commun.* **2018**, *54*, 12483.

[6] X.-Y. Jin, C.-S. Wu, A.-D. Liu, L. Liu, L. Cheng, *Synthesis* **2022**, *54*, 1643.

[7] a) H. Maeda, T. Maeda, K. Mizuno, *Molecules* **2012**, *17*, 5108; b) Y.-P. Ou, J. Zhang, Y. Hu, J. Yin, C. Chi, S. H. Liu, *Dalton Trans.* **2020**, *49*, 16877.

[8] K. Gao, L. Xiao, Y. Kan, L. Li, Y. Yan, H. Huang, J. Peng, Y. Cao, X. Peng, *Chin. J. Chem.* **2016**, *34*, 353.

[9] C. G. Hatchard, C. A. Parker, *Proc. R. Soc. Lond. A* **1956**, *235*, 518.

[10] K. Stranius, K. Börjesson, *Sci. Rep.* **2017**, *7*, 41145.

[11] T. J. B. Zähringer, N. Lopez, R. Schulte, M. Schmitz, H. Ihmels, C. Kerzig, *Angew. Chem. Int. Ed.* **2024**, e202414733.

[12] T. J. B. Zähringer, M. Wienhold, R. Gilmour, C. Kerzig, *J. Am. Chem. Soc.* **2023**, *145*, 21576.

[13] A. M. Helms, R. A. Caldwell, *J. Am. Chem. Soc.* **1995**, *117*, 358.

[14] C. Petermayer, S. Thumser, F. Kink, P. Mayer, H. Dube, *J. Am. Chem. Soc.* **2017**, *139*, 15060.
